# Chemical Proteomics-Guided Identification of a Novel Biological Target of the Bioactive Neolignan Magnolol

**DOI:** 10.3389/fchem.2019.00053

**Published:** 2019-02-08

**Authors:** Chiara Cassiano, Roberta Esposito, Alessandra Tosco, Agostino Casapullo, Matteo Mozzicafreddo, Corrado Tringali, Raffaele Riccio, Maria Chiara Monti

**Affiliations:** ^1^Dipartimento di Farmacia, Università degli Studi di Salerno, Fisciano, Italy; ^2^Scuola di Bioscienze e Medicina Veterinaria, Università degli Studi di Camerino, Camerino, Italy; ^3^Dipartimento di Scienze Chimiche, Università degli Studi di Catania, Catania, Italy

**Keywords:** chemical proteomics, bioactive neolignans, molecular docking, drug affinity responsive target stability, nuclear import

## Abstract

Understanding the recognition process between bioactive natural products and their specific cellular receptors is of key importance in the drug discovery process. In this outline, some potential targets of Magnolol, a natural bioactive compound, have been identified by proteomic approaches. Among them, Importin-β1 has been considered as the most relevant one. A direct binding between Magnolol and this nuclear chaperone has been confirmed by DARTS and molecular docking, while its influence on Importin-β1 translocation has been evaluated by *in vitro* assays.

## Introduction

**Figure 1:**
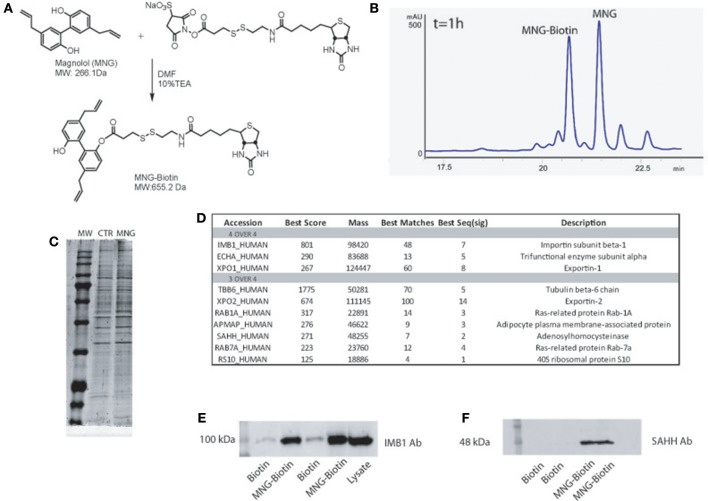
**(A)** Chemical structure of MNG and its reaction with the biotin containing reactive linker. **(B)** HPLC profile of MNG after 1 h of coupling reaction with biotin activated compound. **(C)** SDS-PAGE of proteins fished out by MNG and control. **(D)** MNG targets identified in four or three over four independent AP-MS experiments, reported together with the best Mascot protein Score, best Peptide Matches (number of MS/MS spectra matched to the protein) and best Seq (sig) as the number of significant distinct sequence matches in the protein identification process (see SI for more details); **(E,F)** Western blot analysis on fished out proteins coming from two independent AP-MS experiments using antibodies against Importin β1subunit and SAHH.

The discovery of the biological targets of bioactive natural products is a significant step in chemical biology to point out their mechanism of action (Ziegler et al., 2013). Ongoing our studies on products from natural environment bearing pharmacological effects (Margarucci et al., 2010; Cassiano et al., 2014,a), we were attracted by the chemical architecture of Magnolol (MNG, Figure 1), a dimeric phenolic neolignan endowed with a wide spectrum of biological activities (Lee et al., 2011). Many phenolic compounds are usually engaged in cosmetic, agrofood, and medicinal applications as chemo-preventive agents for their antifungal, anticancer, antioxidant, and hepato-protective functions. Neolignans are the main active components of *Magnolia* genus, used in Chinese and Japanese traditional medicine to counteract anxiety, allergies and gastrointestinal disorders (Shen et al., 2010). Among them, MNG has been isolated from *Magnolia officinalis* and *Magnolia obovata* and it is well-known to exert antitumor, anti-angiogenic, antimicrobial, antiviral, hepato-protective, anti-oxidant, and anti-inflammatory activities (Lee et al., 2011). In literature, several potential MNG targets have been revealed through a non-systematic approach: for instance, MNG is reported to inhibit the growth of non-small cell lung cancer by inhibition of microtubule polymerization (Shen et al., 2017) and, recently, tankyrase-2 (TNKS2), casein kinase 2 (CK2) and bromodomain 9 (Brd9) have been pointed out as its possible targets by *in silico* inverse virtual screening (Di Micco et al., 2018). Nevertheless, an evaluation of its entire interactome in a complex and unbiased protein mixture is still neglected, leaving open questions on its potential multi-target interactions explaining the wide bio-pharmacological properties. On this basis, we applied a combination of mass spectrometry (MS)-based proteomic approaches to define MNG comprehensive interactome, based on a fishing for partners strategy in pseudo-physiological conditions. In particular, our plan consisted in the combination of two different methods, (a) the well-established affinity chromatography, nano-LCMSMS analysis (AP-MS) followed by a database proteins identification using Mascot software to get the specific interactors from a cell lysate by use of the MNG biotinylated derivative, and (b) the drug affinity responsive target stability (DARTS) procedure, which avoids chemical modification of the natural product (Lomenick et al., 2009; Pai et al., 2015; Morretta et al., 2017; del Gaudio et al., 2018). The combination of AP-MS and DARTS is an ideal methodology for a comprehensive identification of the small molecule biological partners, also providing evidence for a direct interaction between the counterparts. Here, few MNG potential partners have been identified and, among them, Importin-β1 has been selected as a novel interesting one: MNG influence on its target has also been demonstrated by *in vitro* experiments.

## Materials and Methods

### Magnolol Coupling to Biotin Linker

2 μmol of MNG (TCI Europe, Zwijndrecht, Belgium) and 20 μmol of Sulfo-NHS activated S-S biotin were solubilized in DMF, 10% tri-ethyl-amine and left 1 h under shaking at r.t. Product formation has been monitored using an Agilent 1100 binary pump, by injecting 20 μL of reaction mixture at different reaction times. A Phenomenex column (Luna Omega Polar C18 150X2.1 mm 5 μm 100A) has been employed as stationary phase and elution has been achieved at a flow rate of 0.2 mL/min by means of a linear gradient of buffer B from 10 to 95% in 25 min (A = 100% H_2_O and 0.1% TFA and B = 95% ACN, 5% H_2_O and 0.1% TFA); chromatograms were recorded at 220, 254, and 280 nm. Products were analyzed by mass spectrometry, employing a QToF Premiere (Waters Co., Milford, USA) equipped with an ESI source. MNG-Biotin was subsequently purified from reaction mixture using the same chromatographic conditions. Sulfo-NHS activated S-S biotin was deactivated with ethanolamine to obtain a control linker for the affinity chromatography experiments. Briefly, 15 μmol of ethanolamine has been conjugated with 15 μmol Sulfo-NHS activated S-S biotin. Adduct formation has been monitored by mass spectrometric analysis with QToF Premiere (Waters Co.).

### Affinity Purification of Magnolol Interactors

HeLa cells were grown in Dulbecco's modified Eagle medium (DMEM) supplemented with 10% (v/v) fetal bovine serum, 100 U/ml penicillin, 100 mg/ml streptomycin, at 37°C in a 5% CO_2_ atmosphere (all reagents were from Euroclone). Cells were collected by centrifugation (600g, 5 min) and washed twice with PBS. Cellular extracts were obtained by glass tight-fitting Dounce manual homogenizations, re-suspending cells pellets in ice cold PBS (137 mM NaCl, 2,7 mM KCl, 10 mM Na_2_HPO_4_, 2 mM KH_2_PO_4_) containing 0.1% Igepal and a protease inhibitor cocktail (Sigma-Aldrich). The resulting suspension has been centrifuged at 10,000 g at 4°C for 10 min to remove cellular debris. By means of Bradford assay, protein concentration has been determined and adjusted to 1 mg/ml. MNG-biotin adduct (10 nmol) and the same amount of the control linker were separately incubated with 1 mg of HeLa derived proteins. Solutions were rotary shaker for 1 h at 4°C. 100 μL of streptavidin-modified beads (Thermo Fisher Scientific, USA) were subsequently added to the protein mixtures. After 1 h at 4°C, unbound proteins were removed and streptavidin beads were collected and extensively washed with 5 ml of PBS (pH 7.4). The bound proteins were finally eluted by boiling beads with 35 μL of SDS-PAGE loading buffer (100 mM Tris pH 6,8, 4% SDS, 0.2% Bromophenol Blue, 20% glycerol, 2% β-mercaptoethanol) and separated on 12% SDS-PAGE. Resulting gel was stained with a Coomassie solution (Thermo Fisher Scientific). SDS-PAGE gel lanes were cut in pieces and digested *in situ* as described by Shevchenko et al. (2006). Briefly, each slice has been reduced with 10 mM 1,4-dithiothreitol (DTT) and alkylated with 54 mM iodoacetamide. Reagents excess has been removed by washing gel pieces with ammonium bicarbonate (50 mM pH 8.5) and CH_3_CN. A 12 ng/mL trypsin solution was added to pieces and rehydration was allowed in ice for 1 h. Afterwards, trypsin excess was discarded and proteins digestion was carried out by incubating gel pieces with 30 μL of ammonium bicarbonate at 37°overnight. The supernatants were collected and peptides were extracted from slices using 100% CH_3_CN. Both supernatants were combined and dried *in vacuo*. The peptide samples were diluted in 10% formic acid before MS analysis. 5 μL of each sample were injected into a nano-ACQUITY UPLC system (Waters) equipped with a 1.7 mm BEH C18 column (Waters). Peptides were separated at a flow rate of 300 nL/min in a 60 min gradient of B (solution A: 95 % H_2_O, 5 % CH_3_CN, 0.1% FA; solution B: 95 % CH_3_CN, 5 % H_2_O, 0.1 % FA); 15–50% B). MS and MS/MS data were acquired on a LTQ Orbitrap XL mass spectrometer (Thermo Scientific). A data dependent acquisition was performed to select and fragment the 10 most intense doubly and triply charged ions. MS data were processed by MS-converter software to generate mgf files for protein identifications. The Mascot server (http://www.matrixscience.com/) was used for database searches. The SwissProt database (release 2017 01, 553,74 sequences, 198,069,095 residues) was employed allowing two missed cleavages, a peptide tolerance of 30 ppm; MS/MS tolerance of 0.8 Da. Carbamidomethyl (C) was set as fixed modification whereas oxidation (M) and phosphorylation (ST) as variable modifications.

### Immunoblotting Assay

Proteins eluted from the above-described experiments were revealed by Western blotting. Samples were run on a 12% SDS-PAGE gel and transferred onto a nitrocellulose membrane. The membrane was incubated for 1 h in a blocking solution containing 25 mM Tris pH 8, 125 mM NaCl, 0.05% Tween20, 5% non-fat dried milk and incubated overnight at 4°C with a polyclonal antibody raised against Importin-β1 and/or SAHH (1:100 in 5% milk; Genetex). Then, the membrane was incubated for 1 h with an anti-rabbit peroxidase-conjugated secondary antibody (1:5000; Thermo Scientific). Importin-β1 and SAHH were detected by using the enhanced chemiluminescence detection system LAS4000 (GE Healthcare, Little Chalfont, UK).

### Drug Affinity Responsive Target Stability (DARTS)

Proteins were extracted from HeLa cells as previously described. MNG was added to lysate samples at different final concentrations (1, 10, and 100 μM); 1% DMSO was used as vehicle and added to control samples. After 1 h at 4°C, samples have been treated with subtilisin (defect 1:500 w:w) and the limited proteolysis was allowed for 45 min at room temperature. PMSF (phenylmethylsulfonyl fluoride) at 1 mM as final concentration was added to quench the digestion. SDS loading buffer was added and 20 μg of each sample were separated by a 12% SDS-PAGE and subjected to immunoblotting. Nitrocellulose membrane has been incubated with the primary antibody specific for Importin β-1 and SAHH (1:100 in 5% milk; Genetex) and subsequently with a primary antibody against GAPDH (1:1000 in 5% milk; Invitrogen) as a loading normalizer.

### Molecular Docking Analysis of the MNG/Importin β1 Complex

Molecular docking analysis has been performed to predict the possible binding mode and the binding strength between MNG, designed and minimized using Avogadro software (Hanwell et al., 2012) as previously reported (Mozzicafreddo et al., 2015), and the human Importin β-1 (using the crystallographic structure with 1QGR as pdb code by Cingolani et al., 1999). Autodock Vina (version 1.1.2, Trott and Olson, 2010), on an Intel Core i7/Mac OS X 10.13 – based platform, has been employed considering a docking zone including the entire protein with a grid of 74, 75 and 71 in the x, y, and z directions, whereas the NNscore 2.0 python script (Durrant and McCammon, 2010) was used to calculate the predicted equilibrium dissociation constant (*K*_*D, pred*_). The final complex geometry was rendered by PyMol software (The PyMOL Molecular Graphics System, Version 2.0.4 Schrödinger, LLC.) whereas the 2D representation was created using PoseView server (Stierand et al., 2006).

### Isolation of Nuclear and Cytosolic Fraction

HeLa cells were seeded in 100 mm tissue culture dishes in Dulbecco's Minimal Essential Medium (DMEM, Euroclone), supplemented with 10% (v/v) fetal bovine serum (Euroclone), 100 U/mL penicillin and 100 g/L streptomycin (Euroclone) at 37°C in a 5% CO_2_ atmosphere. After 24 h, cells were treated or not with 100 μM MNG for 16 h. Then, the cells were scraped and collected by centrifugation at 1000 x *g* and each pellet was suspended in 500 μl of Buffer A (10 mM Hepes pH 7,9; 1 mM EDTA pH 8; 60 mM KCl; 0.2% NP-40; 1 mM DTT; 1 mM PMSF and protease inhibitor cocktail). The cell suspension was passed through a 26-gauge needle 10 times and left on ice for 30 min. Then, the samples were centrifuged at 660*g* for 5 min at 4°C. The pellet contained nuclei and the supernatant contained cytoplasm, membrane and mitochondria. The supernatant was transferred into a fresh tube, 50 μl of Buffer C (250 mM Tris-HCl pH 7,8; 60 mM KCl; 1 mM DTT; 2 mM PMSF; 20% w/v glycerol) were added and the sample was centrifuged at 9,500 g for 15 min at 4°C to obtain the supernatant containing the clear cytosolic fraction. The nuclear pellet was washed three times with 300 μl of Buffer B (10 mM Hepes pH 7,9; 1 mM EDTA pH 8; 60 mM KCl; 1 mM DTT; 1 mM PMSF and protease inhibitor cocktail), suspended in 50 μl of Buffer C and exposed to freezing–thawing cycles for three times. The samples were then centrifuged at 9,500 g for 15' at 4°C and the supernatant containing the nuclear fraction was collected. The protein concentration of cytosolic and nuclear fraction was measured using a Bradford assay.

## Results

The experimental route was arranged in the following steps: (a) production of a biotin-tagged MNG, incubation with HeLa cell lysate and recovery of the specific partners, (b) detection of MNG interactome by high-resolution mass spectrometry and bioinformatics, (c) results validation by DARTS and *in vitro* assays.

### Generation of Mono-Biotinylated MNG Adduct

Initially, MNG has been treated with a N-hydroxysuccinimide activated S–S biotin linker taking advantage of its nucleophilic OH, affording the MNG–biotin derivative in Figure 1A. The reaction was monitored by HPLC-UV (peak at 20.6 min, Figure 1B), by MS (MW of 655.2 Da) and by tandem MS (see Supplementary Figure 1), and then it was tuned employing different molar ratios of the reagents to give 50% yield, after 3 h of reaction. Then, MNG-biotin adduct was purified by HPLC-UV before its use in the proper homogenous affinity chromatography step.

### Identification of MNG Interactome by nano-LC-MSMS Analysis and Immunoblotting

MNG-biotin adduct and free biotin as control were separately incubated with HeLa cell protein extracts for 1 h at 4°C, to allow contacts between the natural compound and its potential partner(s). Then, the complexes between MNG–biotin and its interacting proteins were fished out using streptavidin-bearing solid beads (Margarucci et al., 2015). The matrix beads were washed with phosphate buffer (PBS) to reduce the amount of non-specific ligands, weakly absorbed to the solid matrix. The proteins specifically bound to the beads bearing MNG were released using a highly denaturant buffer, compatible with the subsequent 1D-SDS-PAGE analysis. The protein mixtures eluted from MNG-bearing and control beads were resolved by SDS-PAGE and the corresponding entire gel lanes were divided into several pieces and subjected to an *in situ* trypsin digestion protocol (Shevchenko et al., 2006). The tryptic peptide mixtures coming from each gel slice were then analyzed through nano-flow RP-HPLC MS/MS, and the MS/MS data and, once converted into peak lists, were submitted to Mascot software analysis for protein identification. The list of the MNG potential interactors has been refined as follow: all proteins identified in a single AP-MS experiment with a Mascot score higher than 60 and fully absent in the corresponding control experiment were considered reliable; the four lists obtained by four independent AP-MS experiments were matched as reported in the Venn diagram (Supplementary Table 1 and Supplementary Figure 2) and a more restricted and confident list of putative specific MNG interactors has been obtained as reported in Figure 1C. Importin β-1 and Exportin-1 were recognized as main partners of MNG, on the basis of the highest Mascot score reported in the identification process (Figure 1C and Supplementary Figure 3) together with the trifunctional enzyme subunit alpha, a poorly studied subunit of the mitochondrial fatty acid beta-oxidation multi-enzymatic complex. Moreover, several putative partners of the natural compound were identified in three over four independent experiments such as, for instance, Tubulin isoform 6, Exportin-2 and Adenosylhomocysteinase protein (SAHH). These proteins can be considered conceivable MNG partners: for instance, it is already known that MNG inhibits the growth of non-small cell lung cancer inhibiting microtubule polymerization by its interaction with Tubulin (Shen et al., 2017). The MS-based results were validated by immuno-blotting analysis, using Importin β-1 subunit and SAHH detecting antibody. As reported in Figure 1D, a significant antigen/antibody recognition has been appreciated in the MNG gel lanes compared to the control ones, providing a clear indication of a specific enrichment of both Importin β-1 and SAHH, due to the interaction with the natural compound.

### Application of DARTS Protocol on MNG Target Protein

**Figure 2:**
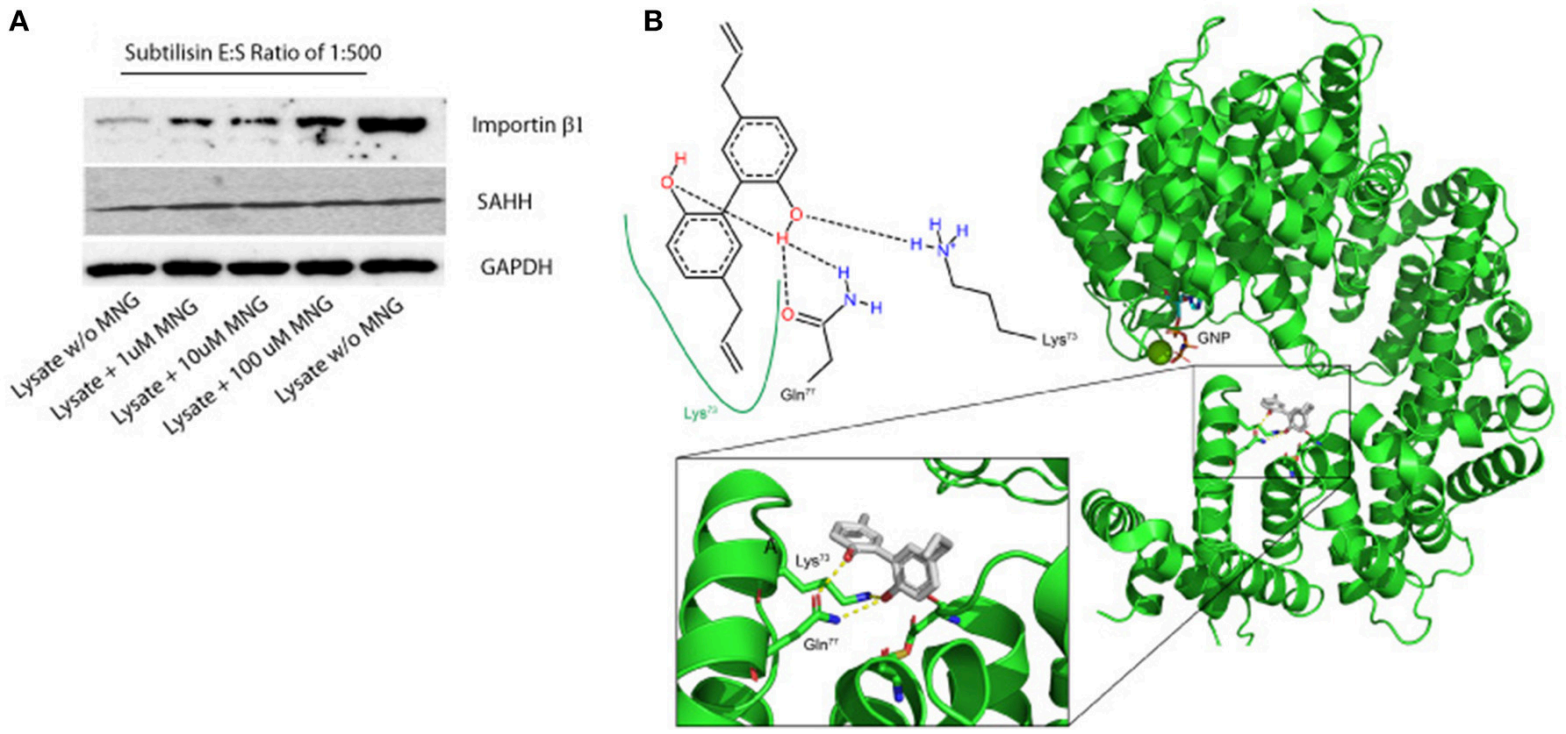
**(A)** Importin β-1 and SAHH protection to subtilisin upon MNG interaction: DARTS was performed using HeLa cell lysates treated or not with increasing quantities of unmodified MNG and samples were detected by immunoblotting. GAPDH served as loading control. Two samples of cell lysates were treated with DMSO and without MNG and only one of them with subtilisin, representing the control experiments. **(B)** Best feasible predicted binding geometry resulted by the molecular docking analysis between Importin β-1 and MNG. Residues involved in the complex formation are shown as stick representation and in the 2D scheme. A zoom-in box has been reported.

An additional evidence of the above results has been obtained when increasing quantities of unmodified MNG were incubated with samples of HeLa cell lysate and then submitted to limited proteolysis with subtilisin, to perform a drug affinity target stability (DARTS) experiment. DARTS strategy is based on the ability of a small molecule to modulate the susceptibility of its target proteins toward the action of proteases. On this starting point, once the cell lysate has been exposed to the small molecule and treated with an unspecific proteolytic enzyme, information on the potential specific targets of the small molecule can be raised evaluating the ability of the compound itself to protect against proteolytic cleavages. As previously described (Lomenick et al., 2009; Pai et al., 2015; Morretta et al., 2017; del Gaudio et al., 2018), it is possible to monitor the protease sensitivity of a particular protein through a semi-quantitative analysis by immunoblotting. Thus, all the samples treated or not with MNG and subtilisin were submitted to SDS-PAGE and immunoblotted using antibodies specific for Importin β-1 and SAHH, separately. As shown in Figure 2A, MNG undoubtedly protects Importin β-1 from the enzymatic proteolytic action, as emerges comparing samples containing increasing amounts of MNG with the control ones, both exposed or not to subtilisin (first and last lane of the gel, respectively). This result gave also evidence of a direct interaction between MNG and Importin β-1.

On the contrary, this effect is not prominent on SAHH protein which is not affected by the presence of MNG. Thus, a more deep characterization of MNG-Importin β-1 complex has been carried out.

### Molecular Docking Analysis of the MNG/Importin β-1 Complex

In parallel, molecular docking analysis of MNG on Importin β-1 has been performed using the three-dimensional structure of human protein whose crystallographic structure has already been resolved (1QGR as pdb code by Cingolani et al., 1999). On the basis of the predicted affinity, MNG produced its best interaction poses into the protein binding site involving the amino acids Lys73, Gln77, Cys118, and Asp160 (Figure 2B). The predicted equilibrium dissociation constant (K_D, pred_) related to this binding site was of 0.43 ± 0.12 μM. In particular, MNG shows feasible interactions with the residues Lys73 (a H-bond and a hydrophobic contact) and Gln77 (two H-bonds) as also reported in the bidimensional scheme of the Figure 2B. A parallel molecular docking analysis has been carried out on another abundant neolignan found in *Magnolia* sp., called Honokiol, whose structure is very close to MNG. The same binding site and a similar K_D, pred_ of 0.22 ± 0.05 μM has been found, suggesting that Importin β-1 could be bound by several biphenols through intramolecular interactions like hydrogen bonding and hydrophobic contacts (Supplementary Figure 4).

Since all the residues involved in Importin β-1-MNG interaction fit in to the so called “contact area I' which manages the interaction between Importin β-1 and the GTP binding protein Ran (Vetter et al., 1999), we were intrigued to gather some proofs on the effect of MNG on this pathway. In fact, the GTP binding protein Ran is involved into cargos release from Importin β-1 and to its subsequent translocation from nucleus to cytoplasm.

### Biological Evaluation of MNG Action on Importin β-1 Localization

**Figure 3:**
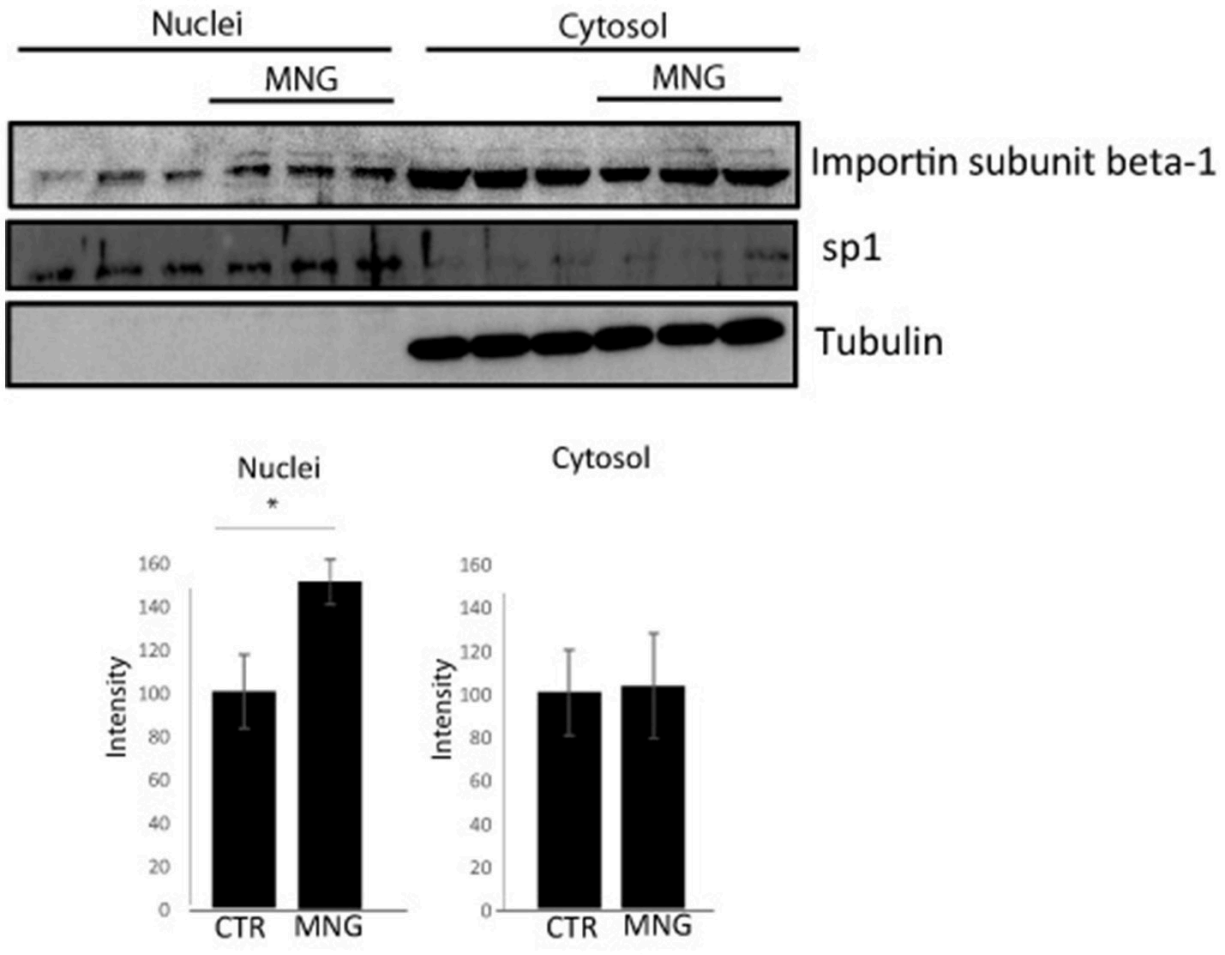
Immunoblotting analysis of nuclear and cytosolic proteins of HeLa cells upon MNG treatment, revealed by an antibody against Importin β1; Transcription factor Sp1 and tubulin served as a loading control to assess the effective nuclei/cytosol separation and for densitometry analysis. The protein levels were quantified using the software IMAGEJ in three independent experiments and results ± SD have been reported in the histograms, setting as 100% the intensity of the Importin β1 signals in control experiments. Asterisk “^*^” represents significant differences among treatments (*p* < 0.05).

The import of proteins usually involves the formation of a heterodimer of Importin α-2 and β-1 subunits. Importin α-2 binds the nuclear signal-containing cargos in the cytoplasm whereas Importin β-1 docks the complex at the cytoplasmic side of the nuclear pore complex. In presence of stimuli, the complex moves into the nuclear pore and the Importin αβ complex dissociates releasing their cargos inside the nucleus upon the binding to a GTP containing Ran protein. Then, GTP-Ran bound to Importin β-1 promotes its translocation into the cytoplasm thanks to GTP hydrolysis (Tran et al., 2014). In this scenario, the experimental conditions to analyze MNG action were first set monitoring cell viability after 16 h of incubation with different concentrations of MNG (from 10 to 100 μM). Since MNG didn't affect cell viability (Supplementary Figure 5), the following experiments were performed incubating HeLa cells for 16 h with MNG at 100 μM. The potential influence of MNG on Importin β-1 localization levels in the cell compartments has been analyzed by western blot on separated nuclear and cytosolic extracts. Figure 3 shows that, upon MNG treatment, Importin β-1 levels increased in nuclear extracts, suggesting that the interaction of MNG with this protein could influence its affinity to the GTP binding protein Ran and thus modulate Importin β-1 localization.

## Conclusions

The exploration of the interactions occurring between natural products, whose bioactivity is well-proved in traditional eastern medicine, and their cellular receptors is meaningful. Here, some potential targets of Magnolol, a neolignan mainly found in the bark of *Magnolia officinalis*, have been acknowledged by a combination of proteomic strategies and, among them, Importin-β1 has been validated by DARTS and immunoblotting approaches. Furthermore, molecular docking analysis pointed out that Magnolol and other neolignans, such as Honokiol, interacts within Importin-β1 in its contact area with the GTP binding protein Ran, deputed to the translocation of Importin-β1 into cytoplasm. Finally, the activity of the natural compound on the translocation of this nuclear chaperone has been evaluated by *in vitro* assays. Although it is not possible to exclude that an alteration in protein translocation might also be caused by differences in protein turnover and half-life, it seems clear that Importin β-1 levels increased in nuclear extracts upon Magnolol treatment, proving that the supposed interaction modulates Importin β-1 localization.

Importin-β1, also called karyopherin β-1 (kpnβ-1) as a member of the karyopherin β superfamily (Moroianu, 1998), is a very attractive target since it takes part in nuclear proteins import, either in connection with an adapter protein, like an Importin-α subunit, or by working as autonomous nuclear transport receptor. Indeed, several million proteins are continuously transported between the nucleus and cytoplasm by receptor mediated transfer. This bidirectional traffic goes on through nuclear pore complexes and it is mainly due to shuttling proteins as Importins and Exportins. Numerous import and export factors are present in the cell, but the most important ones are Transportin-1, the Importin α2/β-1 complex and Exportin-1. In most cases, the cytoplasm to nucleus transport is coordinated by the small GTPase Ran proteins, which bind to the transport receptors into the nuclei, promoting the cargos release and the subsequent reverse of the transport proteins into the cytoplasm (Pemberton and Paschal, 2005).

In particular, Importin β-1 action is associated with the occurrence of several disease. In this perspective, if one transport pathway is activated or repressed, the multiple cargoes are relocated within the cell, and then the physiological state of the cell is altered. In particular, Importin β-1 action is linked to tumors development (Çagatay and Chook, 2018) and to several neurodegenerative disorders (Boeynaems et al., 2016). Definitely, the altered expression or translocation of this protein should affect the localization of multiple cargoes, and some of them participate in oncogenesis (Kimura and Imamoto, 2014). For instance, it has been demonstrated that the inhibition of Crm1 and Importin-β1 in cervical cancer cells reduced cell proliferation, making both proteins promising candidates as both biomarkers and potential anticancer therapeutic targets (van der Watt et al., 2009). Moreover, modification in the subcellular localization of transcription factors such as Importin-β1 has been detected in neurons affected by neurodegenerative diseases such as Parkinson, Alzheimer, amyotrophic lateral sclerosis and polyglutamine diseases. Oxidative stress, with a pathogenic function in these syndromes, has also been connected with alterations in nuclear transport. In fact, oxidative modifications can alter the cytoplasmic to nuclear ratios of proteins by regulating interactions with cytoplasmic anchors and masking or exposing nuclear import and export signals (Patel and Chu, 2011).

Thus, the discovery of natural metabolites affecting Importin β-1 cellular localization could be of wide interest both as a potential lead compounds for the above mentioned diseases and as biochemical devices. The gathered data, obtained by a multi-disciplinary strategy including chemical proteomics, DARTS, molecular docking and *in vitro* assays, point toward the identification of Importin β-1 as a novel biological interactor of Magnolol.

## Author Contributions

CC, RE, and MM performed the experiments. CT provided the raw materials. AC, RR, and AT conceived and designed the biological assays. MCM conceived and designed the proteomics assays, analyzed the data and wrote the paper.

### Conflict of Interest Statement

The authors declare that the research was conducted in the absence of any commercial or financial relationships that could be construed as a potential conflict of interest.
