# Supplementary material for: Chemical Proteomics-Guided Identification of a Novel Biological Target of the Bioactive Neolignan Magnolol
Source: Front Chem. 2019 Feb 8;7:53. doi: 10.3389/fchem.2019.00053 (PMC6375844; doi:10.3389/fchem.2019.00053)
Supplement: Supplementary file 1 [file Data_Sheet_1.PDF]

Supplementary Material

Identification of a novel biological target of the bioactive neolignan Magnolol guided by chemical proteomics.

Chiara Cassiano,[1] Roberta Esposito,[1] Alessandra Tosco,[1] Agostino Casapullo,[1] Matteo Mozzicafreddo,[2] Corrado Tringali,[3] Raffaele Riccio,[1*] and Maria Chiara Monti[1]*

[1] Dipartimento di Farmacia, Università degli Studi di Salerno, via Giovanni Paolo II 132, 84084 Fisciano (Italy);

[2] Scuola di Bioscienze e Medicina Veterinaria, Università degli Studi di Camerino, via Gentile III da Varano, 62032 Camerino (Italy)

[3] Dipartimento di Scienze Chimiche, Università degli Studi di Catania, Viale Andrea Doria 6, 95131 Catania (Italy)

*** Correspondence:**
Maria Chiara Monti, mcmonti@unisa.it

1 Analysis of MNG reactivity towards N-hydroxysuccinimide activated S–S biotin linker.

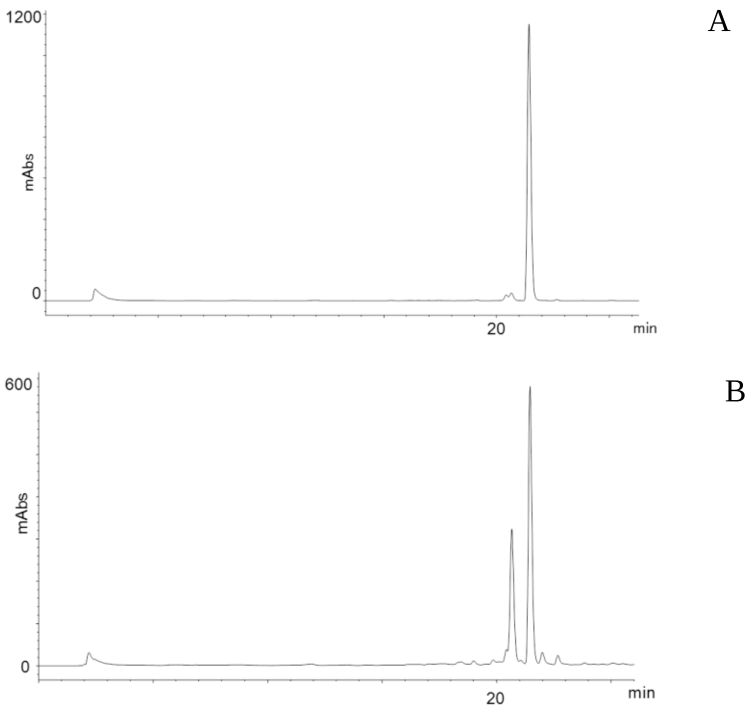

C

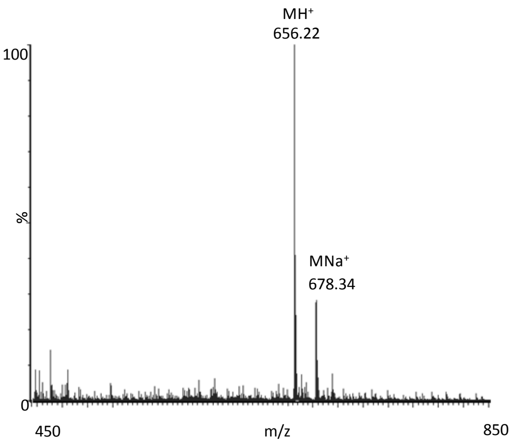

D

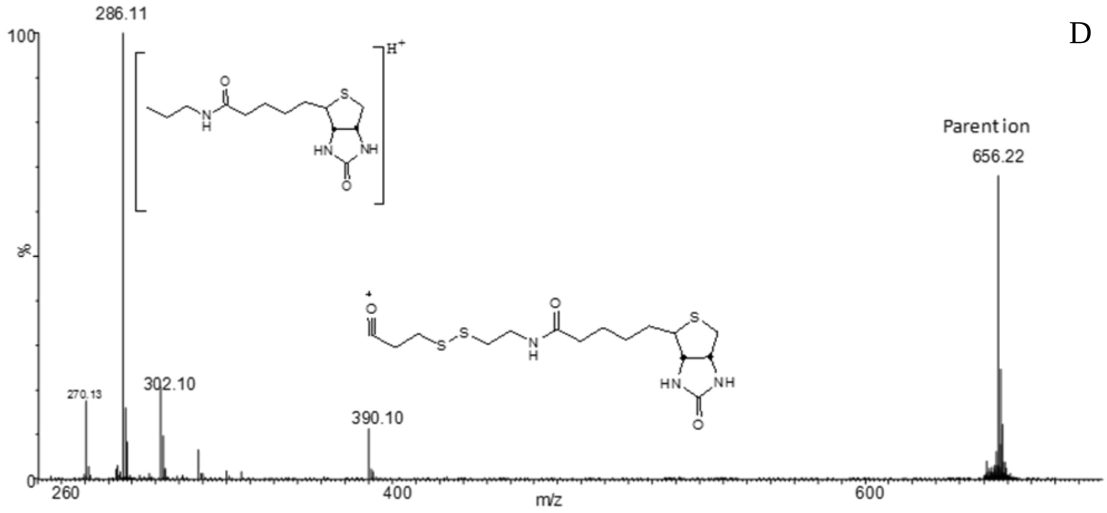

Supplementary Figure 1. Panel A shows the HPLC-UV (at 280 nm) trace of pure MNG in absence of biotin linker. The peak at 22 min contains MNG. Panel B shows the HPLC-UV (at 280 nm) trace of MNG in presence of the biotin linker after 30 min of reaction and the MS spectra of the peak at 21 min is reported in Panel C. Panel C shows the MS spectrum of the MNG-biotin adduct visible as MH^+ and MNa^+ ions. Panel D shows the MS/MS fragmentation of the ion at m/z of 656.2 obtained upon the reaction of MNG with biotin activated linker. The daughter peaks at m/z 390.1 and 286.1 were both attributed to fragments containing the biotin linker.

2. Mascot based identification of MNG partners in four independent experiments.

Accession	Score 1	Matches 1	Score 2	Matches 2	Score 3	Matches 3	Score 4	Matches 4	Description
1433G_HUMAN	x	x	x	x	x	x	462	29	14-3-3 protein gamma
1433Z_HUMAN	x	x	x	x	x	x	570	42	14-3-3 protein zeta/delta
1B35_HUMAN	256	12	x	x	x	x	x	x	HLA class I histocompatibility antigen, B-35 alpha chain
4F2_HUMAN	x	x	70	5	191	10	x	x	4F2 cell-surface antigen heavy chain
AAKG1_HUMAN	x	x	64	2	x	x	x	x	5'-AMP-activated protein kinase subunit gamma-1
ACACA_HUMAN	x	x	x	x	x	x	475	42	Acetyl-CoA carboxylase 1
ACTB_HUMAN	x	x	x	x	1748	100	x	x	Actin, cytoplasmic 1
ACTN4_HUMAN	66	6	x	x	x	x	858	37	Alpha-actinin-4
ADT2_HUMAN	x	x	x	x	x	x	319	22	ADP/ATP translocase 2
ADT3_HUMAN	x	x	x	x	x	x	206	17	ADP/ATP translocase 3
AHNK_HUMAN	x	x	x	x	x	x	183	37	Neuroblast differentiation-associated protein AHNAK
AL3A2_HUMAN	x	x	131	2	x	x	x	x	Fatty aldehyde dehydrogenase
ALBU_HUMAN	x	x	x	x	249	9	73	4	Serum albumin
ALDOA_HUMAN	x	x	x	x	274	8	853	43	Fructose-bisphosphate aldolase A
AN32A_HUMAN	x	x	x	x	x	x	69	2	Acidic leucine-rich nuclear phosphoprotein 32 family A
AN32B_HUMAN	x	x	x	x	x	x	84	5	Acidic leucine-rich nuclear phosphoprotein 32 family B
ANM1_HUMAN	x	x	x	x	x	x	62	1	Protein arginine N-methyltransferase 1
ANX11_HUMAN	x	x	351	11	212	18	x	x	Annexin A11
ANXA1_HUMAN	x	x	x	x	577	17	x	x	Annexin A1
ANXA2_HUMAN	x	x	x	x	x	x	695	49	Annexin A2
ANXA5_HUMAN	x	x	x	x	77	2	1087	46	Annexin A5
APMAP_HUMAN	276	9	x		121	5	84	2	Adipocyte plasma membrane-associated protein
ARL1_HUMAN	x	x	x	x	124	4	x	x	ADP-ribosylation factor-like protein 1
ATPB_HUMAN	x	x	x	x	72	14	x	x	ATP synthase subunit beta, mitochondrial
BAX_HUMAN	x	x	x	x	x	x	98	2	Apoptosis regulator BAX
BZW2_HUMAN	x	x	180	10	x	x	x	x	Basic leucine zipper and W2 domain-containing protein 2
C1TC_HUMAN	x	x	x	x	183	7	x	x	C-1-tetrahydrofolate synthase, cytoplasmic
CALX_HUMAN	x	x	x	x	247	14	85	5	Calnexin
CAND1_HUMAN	x	x	x	x	x	x	197	20	Cullin-associated NEDD8-dissociated protein 1
CAP1_HUMAN	x	x	x	x	x	x	84	4	Adenylyl cyclase-associated protein 1
CAPZB_HUMAN	x	x	x	x	x	x	198	7	F-actin-capping protein subunit beta
CH60_HUMAN	x	x	x	x	430	19	877	35	60 kDa heat shock protein, mitochondrial
CLPX_HUMAN	x	x	71	5	x	x	x	x	ATP-dependent Clp protease ATP-binding subunit clpX-like
COF1_HUMAN	x	x	x	x	263	10			Cofilin-1
COPA_HUMAN	x	x	x	x	78	4	x	x	Coatomer subunit alpha
CYBP_HUMAN	x	x	x	x	69	4	x	x	Calcyclin-binding protein
DNJA1_HUMAN	x	x	x	x	128	7	x	x	DnaJ homolog subfamily A member 1
DX39A_HUMAN	x	x	x	x	x	x	86	7	ATP-dependent RNA helicase DDX39A
ECHA_HUMAN	290	10	64	4	199	13	69	1	Trifunctional enzyme subunit alpha, mitochondrial
ECHB_HUMAN	x	x	104	7	x	x	x	x	Trifunctional enzyme subunit beta, mitochondrial
EF1A1_HUMAN	x	x	x	x	484	24	x	x	Elongation factor 1-alpha 1

EF1A3_HUMAN	x	x	x	x	x	x	206	13	Putative elongation factor 1-alpha-like 3
EF1D_HUMAN	x	x	x	x	x	x	118	3	Elongation factor 1-delta
EF1G_HUMAN	x	x	x	x	128	5	508	19	Elongation factor 1-gamma
EF2_HUMAN	x	x	x	x	225	16	x	x	Elongation factor 2
EFHD2_HUMAN	x	x	x	x	x	x	86	4	EF-hand domain-containing protein D2
ENOA_HUMAN	x	x	x	x	517	18	x	x	Alpha-enolase
ENPL_HUMAN	x	x	x	x	213	13	888	48	Endoplasmin
ERO1A_HUMAN	x	x	x	x	125	2	x	x	ERO1-like protein alpha
ESYT1_HUMAN	78	3	x	x	x	x	x	x	Extended synaptotagmin-1
EZRI_HUMAN	x	x	x	x	x	x	127	8	Ezrin
F10A1_HUMAN	x	x	x	x	x	x	85	4	Hsc70-interacting protein
FAS_HUMAN	x	x	x	x	589	36	585	30	Fatty acid synthase
FILA2_HUMAN	248	7	x	x	x	x	x	x	Filaggrin-2
FLNA_HUMAN	x	x	x	x	70	6	x	x	Filamin-A
FLNB_HUMAN	x	x	x	x	x	x	472	34	Filamin-B
FSCN1_HUMAN	x	x	x	x	x	x	82	7	Fascin
GBB2_HUMAN	x	x	60	2	x	x	x	x	Guanine nucleotide-binding protein G(I)/G(S)/G(T) beta-2
GCN1_HUMAN	86	10	119	15	x	x	x	x	eIF-2-alpha kinase activator GCN1
GDIR1_HUMAN	x	x	x	x	x	x	121	5	Rho GDP-dissociation inhibitor 1
GNAI3_HUMAN	80	2	x	x	x	x	x	x	Guanine nucleotide-binding protein G(k) alpha
GNAT1_HUMAN	x	x	x	x	x	x	189	6	Guanine nucleotide-binding protein G(t) alpha-1
GRP75_HUMAN	x	x	x	x	275	8	758	26	Stress-70 protein, mitochondrial
GRP78_HUMAN	x	x	x	x	185	9	x	x	78 kDa glucose-regulated protein
GSTP1_HUMAN	x	x	x	x	124	5		x	Glutathione S-transferase P
H90B4_HUMAN	x	x	x	x	x	x	240	17	Putative heat shock protein HSP 90-beta 4
HNRPQ_HUMAN	x	x	x	x	x	x	96	3	Heterogeneous nuclear ribonucleoprotein Q
HPCA_HUMAN	x	x	x	x	85	6	x	x	Neuron-specific calcium-binding protein hippocalcin
HS105_HUMAN	x	x	x	x	x	x	141	17	Heat shock protein 105 kDa
HS71A_HUMAN	x	x	x	x	x	x	696	27	Heat shock 70 kDa protein 1A
HS90A_HUMAN	x	x	x	x	1305	66	x	x	Heat shock protein HSP 90-alpha
IDH3A_HUMAN	x	x	134	8	x	x	x	x	Isocitrate dehydrogenase [NAD] subunit alpha
IF4A1_HUMAN	x	x	x	x	x	x	1034	29	Eukaryotic initiation factor 4A-I
IF4G1_HUMAN	x	x	x	x	x	x	130	7	Eukaryotic translation initiation factor 4 gamma 1
IMB1_HUMAN	641	24	519	60	801	48	747	48	Importin subunit beta-1
IMDH2_HUMAN	x	x	x	x	231	12	138	7	Inosine-5'-monophosphate dehydrogenase 2
IPO4_HUMAN	x	x	x	x	66	10	x	x	Importin-4
KPYM_HUMAN	x	x	x	x	x	x	73	15	Pyruvate kinase PKM
LCN1_HUMAN	x	x	x	x	x	x	97	4	Lipocalin-1
LDHB_HUMAN	x	x	x	x	472	18	179	8	L-lactate dehydrogenase B chain
LMAN2_HUMAN	x	x	x	x	x	x	532	17	Vesicular integral-membrane protein VIP36
LMNA_HUMAN	x	x	x	x	x	x	328	15	Prelamin-A/C
MAGA4_HUMAN	x	x	x	x	x	x	283	16	Melanoma-associated antigen 4
MCCA_HUMAN	x	x	577	48	x	x	x	x	Methylcrotonoyl-CoA carboxylase subunit alpha

MDHM_HUMAN	x	x	x	x	x	x	141	7	Malate dehydrogenase, mitochondrial
METK2_HUMAN	x	x	x	x	x	x	82	2	S-adenosylmethionine synthase isoform type-2
MPCP_HUMAN	x	x	60	5	x	x	x	x	Phosphate carrier protein, mitochondrial
MPRI_HUMAN	68	6	x	x	x	x	x	x	Cation-independent mannose-6-phosphate receptor
MYH9_HUMAN	x	x	x	x	x	x	276	32	Myosin-9
NASP_HUMAN	x	x	x	x	x	x	95	3	Nuclear autoantigenic sperm protein
NDK8_HUMAN	x	x	x	x	x	x	139	7	Putative nucleoside diphosphate kinase
NDKA_HUMAN	159	5	x	x	x	x	x	x	Nucleoside diphosphate kinase A
NIBL1_HUMAN	x	x	x	x	x	x	69	2	Niban-like protein 1
NSDHL_HUMAN	x	x	x	x	80	5	x	x	Sterol-4-alpha-carboxylate 3-dehydrogenase
OAT_HUMAN	x	x	x	x	114	4	x	x	Ornithine aminotransferase, mitochondrial
OXDD_HUMAN	x	x	x	x	73	34	62	21	D-aspartate oxidase
PDIA4_HUMAN	x	x	x	x	x	x	640	10	Protein disulfide-isomerase A4
PFD2_HUMAN	x	x	x	x	x	x	98	2	Prefoldin subunit 2
PGAM1_HUMAN	x	x	x	x	x	x	63	2	Phosphoglycerate mutase 1
PGK1_HUMAN	309	19	x	x	x	x	745	47	Phosphoglycerate kinase 1
PGM1_HUMAN	x	x	x	x	x	x	137	3	Phosphoglucomutase-1
PHB2_HUMAN	91	3	x	x	x	x	224	8	Prohibitin-2
PLEC_HUMAN	172	31	633	53	x	x	x	x	Plectin
PPIA_HUMAN	69	8	x	x	x	x	x	x	Peptidyl-prolyl cis-trans isomerase A
PRDX1_HUMAN	x	x	x	x	x	x	927	41	Peroxiredoxin-1
PRDX2_HUMAN	x	x	x	x	758	27	x	x	Peroxiredoxin-2
PRDX4_HUMAN	x	x	x	x	448	29	x	x	Peroxiredoxin-4
PRDX6_HUMAN	x	x	x	x	x	x	726	37	Peroxiredoxin-6
PRKDC_HUMAN	197	27	x	x	189	31	x	x	DNA-dependent protein kinase catalytic subunit
PSA7_HUMAN	x	x	x	x	x	x	180	6	Proteasome subunit alpha type-7
PSA7L_HUMAN	x	x	x	x	74	1	x	x	Proteasome subunit alpha type-7-like
PSD13_HUMAN	x	x	x	x	120	5	x	x	26S proteasome non-ATPase regulatory subunit 13
PSDE_HUMAN	x	x	x	x	77	2	x	x	26S proteasome non-ATPase regulatory subunit 14
PSMD7_HUMAN	x	x	x	x	118	8	x	x	26S proteasome non-ATPase regulatory subunit 7
PSME2_HUMAN	x	x	x	x	x	x	84	3	Proteasome activator complex subunit 2
PSME3_HUMAN	x	x	x	x	x	x	122	3	Proteasome activator complex subunit 3
PUR6_HUMAN	x	x	x	x	x	x	73	4	Multifunctional protein ADE2
PYGB_HUMAN	x	x	x	x	x	x	316	15	Glycogen phosphorylase, brain form
PYR1_HUMAN	x	x	x	x	583	29	x	x	CAD protein
RAB10_HUMAN	x	x	x	x	x	x	311	12	Ras-related protein Rab-10
RAB14_HUMAN	x	x	158	9	x	x	x	x	Ras-related protein Rab-14
RAB1A_HUMAN	300	11	166	5	x	x	317	14	Ras-related protein Rab-1A
RAB5A_HUMAN	70	2	x	x	x	x	x	x	Ras-related protein Rab-5A
RAB6A_HUMAN	x	x	x	x	212	9	244	11	Ras-related protein Rab-6A
RAB7A_HUMAN	128	4	x	x	233	12	186	9	Ras-related protein Rab-7a
RACK1_HUMAN	x	x	x	x	248	14	796	34	Receptor of activated protein C kinase 1
RAN_HUMAN	x	x	x	x	113	3	458	16	GTP-binding nuclear protein Ran
RB11A_HUMAN	x	x	64	1	x	x	x	x	Ras-related protein Rab-11A

RINI_HUMAN	x	x	x	x	x	x	124	2	Ribonuclease inhibitor
RIR2_HUMAN	x	x	x	x	73	2	129	3	Ribonucleoside-diphosphate reductase subunit M2
RL11_HUMAN	x	x	x	x	67	3	x	x	60S ribosomal protein L11
RL14_HUMAN	x	x	x	x	x	x	176	5	60S ribosomal protein L14
RL4_HUMAN	x	x	x	x	x	x	71	9	60S ribosomal protein L4
RL7_HUMAN	x	x	x	x	182	5	184	7	60S ribosomal protein L7
RLA0_HUMAN	150	3	x	x	x	x	x	x	60S acidic ribosomal protein P0
RS10_HUMAN	70	3	x	x	82	4	125	3	40S ribosomal protein S10
RS14_HUMAN	178	7	x	x	x	x	x	x	40S ribosomal protein S14
RS18_HUMAN	x	x	x	x	74	6	x	x	40S ribosomal protein S18
RS2_HUMAN	x	x	x	x	x	x	132	10	40S ribosomal protein S2
RS25_HUMAN	x	x	x	x	75	3	x	x	40S ribosomal protein S25
RS3A_HUMAN	x	x	x	x	x	x	97	7	40S ribosomal protein S3a
RS4X_HUMAN	x	x	x	x	x	x	220	10	40S ribosomal protein S4, X isoform
RS5_HUMAN	202	10	x	x	x	x	x	x	40S ribosomal protein S5
RS7_HUMAN	x	x	x	x	76	2	x	x	40S ribosomal protein S7
RS8_HUMAN	x	x	x	x	x	x	297	14	40S ribosomal protein S8
RS9_HUMAN	x	x	x	x	x	x	118	10	40S ribosomal protein S9
RTN4_HUMAN	x	x	230	10	x	x	x	x	Reticulon-4
RUVB1_HUMAN	x	x	x	x	x	x	98	6	RuvB-like 1
SAHH_HUMAN	87	2	x	x	271	7	62	3	Adenosylhomocysteinase
SC24C_HUMAN	x	x	x	x	66	2	x	x	Protein transport protein Sec24C
SERA_HUMAN	x	x	x	x	1533	46	595	20	D-3-phosphoglycerate dehydrogenase
SETLP_HUMAN	x	x	x	x	x	x	126	4	Protein SETSIP
SKP1_HUMAN	x	x	x	x	126	3	x	x	S-phase kinase-associated protein 1
SRSF1_HUMAN	65	4	x	x	x	x	x	x	Serine/arginine-rich splicing factor 1
SYEP_HUMAN	79	8	x	x	x	x	117	7	Bifunctional glutamate/proline--tRNA ligase
SYMC_HUMAN	x	x	90	5	x	x	x	x	Methionine--tRNA ligase, cytoplasmic
SYYC_HUMAN	77	3	x	x	x	x	x	x	Tyrosine--tRNA ligase, cytoplasmic
TBA1C_HUMAN	x	x	3161	157	x	x	x	x	Tubulin alpha-1C chain
TBB6_HUMAN	x	x	947	39	1775	70	69	3	Tubulin beta-6 chain
TCPB_HUMAN	x	x	x	x	70	8	x	x	T-complex protein 1 subunit beta
TCPD_HUMAN	x	x	x	x	185	8	289	16	T-complex protein 1 subunit delta
TCPE_HUMAN	x	x	x	x	190	8	x	x	T-complex protein 1 subunit epsilon
TCPH_HUMAN	x	x	x	x	107	11	x	x	T-complex protein 1 subunit eta
TCPQ_HUMAN	x	x	x	x	662	21	x	x	T-complex protein 1 subunit theta
TCPZ_HUMAN	x	x	x	x	x	x	192	29	T-complex protein 1 subunit zeta
TFR1_HUMAN	x	x	x	x	74	12	x	x	Transferrin receptor protein 1
TKT_HUMAN	x	x	x	x	x	x	704	50	Transketolase
TLN1_HUMAN	x	x	x	x	x	x	99	8	Talin-1
TM109_HUMAN	x	x	x	x	83	2	x	x	Transmembrane protein 109
TMX1_HUMAN	x	x	210	6	x	x	x	x	Thioredoxin-related transmembrane protein 1
TNPO1_HUMAN	x	x	123	3	x	x	x	x	Transportin-1

TNPO2_HUMAN	74	9	x	x	x	x	125	10	Transportin-2
TPD54_HUMAN	x	x	x	x	69	1	229	10	Tumor protein D54
TPM3_HUMAN	x	x	x	x	x	x	522	39	Tropomyosin alpha-3 chain
TRAP1_HUMAN	x	x	x	x	x	x	666	20	Heat shock protein 75 kDa, mitochondrial
TWF1_HUMAN	x	x	x	x	x	x	203	7	Twinfilin-1
TXLNA_HUMAN	x	x	x	x	x	x	239	7	Alpha-taxilin
VIGLN_HUMAN	64	4	x	x	x	x	x	x	Vigilin
VIME_HUMAN	x	x	x	x	x	x	264	22	Vimentin
XPO1_HUMAN	238	15	129	14	105	14	267	20	Exportin-1
XPO2_HUMAN	390	70	x	x	674	100	271	44	Exportin-2
XPO5_HUMAN	138	5	x	x	x	x	x	x	Exportin-5
XRCC5_HUMAN	x	x	x	x	x	x	207	13	X-ray repair cross-complementing protein 5

Supplementary Table 1: The proteins identified in four independent MNG fishing for partners experiments with a Mascot score $\geq$ 60 and not identified in the corresponding control experiments are here reported. For each protein the following parameters are reported: Mascot score (Score) and number of matched peptides (Matches).

3. Venn diagram of the MNG interacting partners identified by nano-ESI-LCMSMS analysis

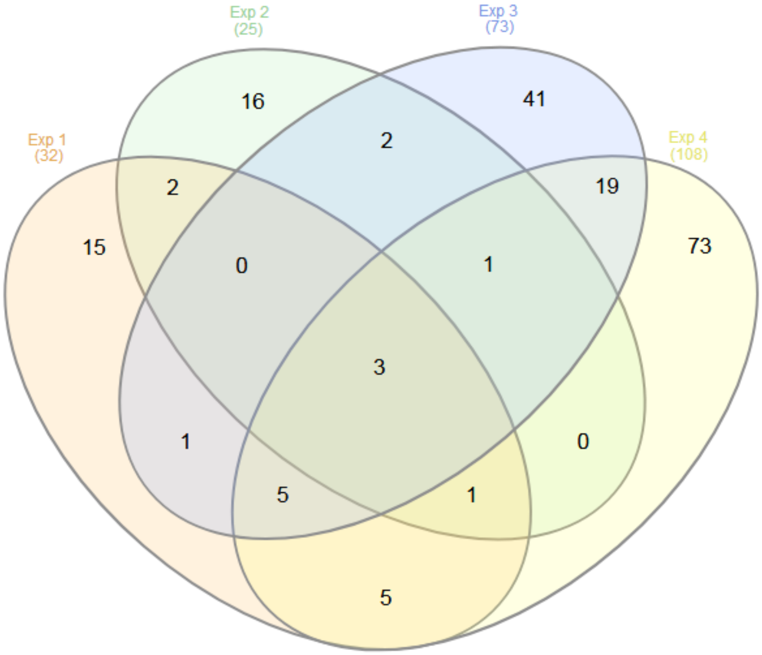

Supplementary Figure 2. The Venn diagrams of MNG interacting partners identified in four independent experiments obtained using InteractiVenn software (http://www.interactivenn.net/).

4. Mascot search results on the MS/MS based identification of Importin β-1.

Exp 1:

MATRIX SCIENCE **MASCOT Search Results**

Protein View: IMB1_HUMAN

Importin subunit beta-1 OS=Homo sapiens GN=KPNB1 PE=1 SV=2

**Database:**	SwissProt
**Score:**	641
**Nominal mass (M_r):**	98420
**Calculated pI:**	4.68
**Taxonomy:**	Homo sapiens

Sequence similarity is available as an NCBI BLAST search of IMB1_HUMAN against nr.

Search parameters

**MS data file:**	C:\Users\Mascott\Desktop\chiara cassisy\MNGFISH\fish23112017\20171122_mng10uM_2.mgf
**Enzyme:**	Trypsin: cuts C-term side of KR unless next residue is P.
**Fixed modifications:**	Carbamidomethyl (C)
**Variable modifications:**	Oxidation (M), Phospho (ST)

Protein sequence coverage: 6%

Matched peptides shown in ***bold red***.

1 MELITILEKT VSPDRLELEA AQKFLER**AAV ENLPTFLVEL SR**VLANPGNS
51 QVARVAAGLQ IKNSLTSKDP DIKAQYQQR**W LAIDANAR**RE VKNYVLQTLG
101 TETYRPSSAS QCVAGIACAE IPVNQWPELI PQLVANVTNP NSTEHMKEST
151 LEAIGYICQD IDPEQLQDKS NEILTAIIQG MRKEEPSNNV KLAATNALLN
201 SLEFTKANFD KESERHFIMQ VVCEATQCPD TRVRVAALQN LVKIMSLYYQ
251 YMETYMGPAL FAITIEAMKS DIDEVALQGI EFWSNVCDEE MDLAIEASEA
301 AEQGRPPEHT SKFYAK**GALQ YLVPILTQTL TKQDENDDDD DWNPCK**AAGV
351 CLMLLATCCE DDIVPHVLPF IKEHIKNPDW RYRDAAVMAF GCILEGPEPS
401 QLKPLVIQAM PTLIELMKDP SVVVRDTAAW TVGRICELLP EAAINDVYLA
451 PLLQCLIEGL SAEPRVASNV CWAFSSLAEA AYEAADVADD QEEPATYCLS
501 SSFELIVQKL LETTDRPDGH QNNLRSSAYE SLMEIVKNSA KDCYPAVQKT
551 TLVIMERLQQ VLQMESHIQS TSDRIQFNDL QSLLCATLQN VLRKVQHQDA
601 LQISDVVMAS LLRMFQSTAG SGGVQEDALM AVSTLVEVLG GEFLKYMEAF
651 KPFLGIGLKN YAEYQVCLAA VGLVGDLCRA LQSNIIPFCD EVMQLLLENL
701 GNENVHRSVK PQILSVFGDI ALAIGGEFKK YLEVVLNTLQ QASQAQVDKS
751 DYDMVDYLNE LRESCLEAYT GIVQGLKGDQ ENVHPDVMLV QPRVEFILSF
801 IDHIAGDEDH TDGVVACAAG LIGDLCTAFG KDVLKLVEAR PMIHELLTEG
851 RRSKTNKAKT LATWATKELR KLKNQA

MS/MS Fragmentation of **QDENDDDDDWNPCK**
Found in **IMB1_HUMAN** in **SwissProt**, Importin subunit beta-1 OS=Homo sapiens GN=KPNB1 PE=1 SV=2

Match to Query 2137: 1764.618504 from(883.316528,2+) intensity(112313.1016) rtinseconds(2149.6899) index(1431)
Title: 20171122_mng10uM_2.2057.2057.2 File:"20171122_mng10uM_2.raw", NativeID:"controllerType=0 controllerNumber=1 scan=2057"
Data file C:\Users\Mascott\Desktop\chiara cassisy\MNGFISH\fish23112017\20171122_mng10uM_2.mgf

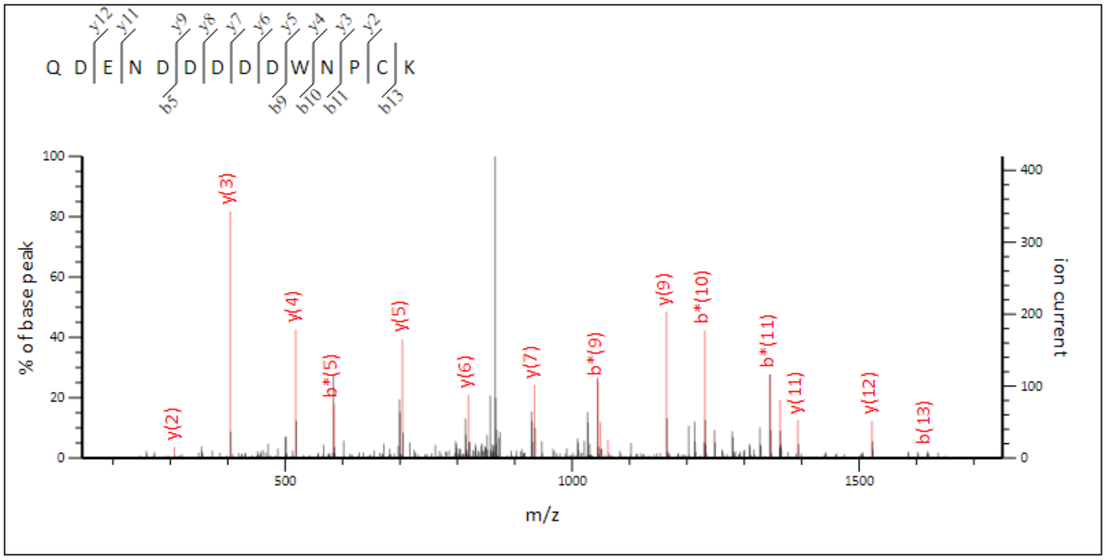

MS/MS Fragmentation of **WLAIDANAR**
Found in **IMB1_HUMAN** in **SwissProt**, Importin subunit beta-1 OS=Homo sapiens GN=KPNB1 PE=1 SV=2

Match to Query 411: 1028.539770 from(515.277161,2+) intensity(1175269.3750) rtinseconds(2820.5761) index(2141)
Title: 20171122_mng10uM_2.2814.2814.2 File:"20171122_mng10uM_2.raw", NativeID:"controllerType=0 controllerNumber=1 scan=281
Data file C:\Users\Mascott\Desktop\chiara cassisy\MNGFISH\fish23112017\20171122_mng10uM_2.mgf

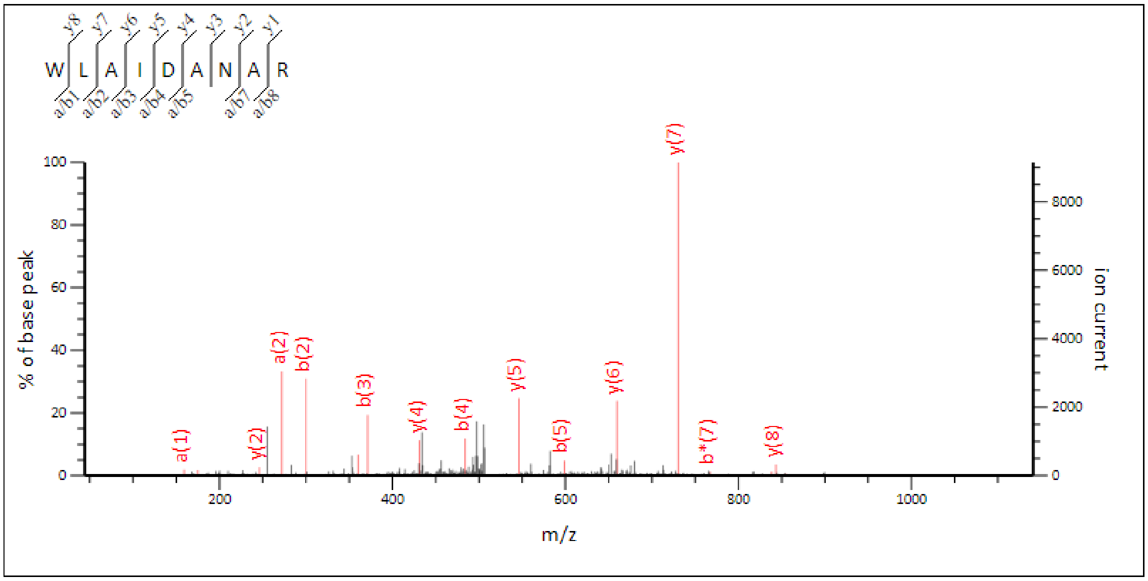

Exp 2:

MATRIX SCIENCE MASCOT Search Results

Protein View: IMB1_HUMAN

Importin subunit beta-1 OS=Homo sapiens GN=KPNB1 PE=1 SV=2

**Database:**	SwissProt
**Score:**	519
**Nominal mass (M_r):**	98420
**Calculated pI:**	4.68
**Taxonomy:**	Homo sapiens

Sequence similarity is available as an NCBI BLAST search of IMB1_HUMAN against nr.

Search parameters

**MS data file:**	C:\Users\Mascott\Desktop\chiara cassisy\magnololo\MNGFISH\FISHING_04032017\20171201_MNG_10uM_2.mgf
**Enzyme:**	Trypsin: cuts C-term side of KR unless next residue is P.
**Fixed modifications:**	Carbamidomethyl (C)
**Variable modifications:**	Oxidation (M), Phospho (ST)

Protein sequence coverage: 15%

Matched peptides shown in ***bold red***.

1 MELITILEKT VSPDRLELEA AQKFLER**AAV ENLPTFLVEL SR**VLANPGNS
51 QVARVAAGLQ IKNSLTSKDP DIKAQYQQR**W LAIDANAR**RE VKNYVLQTLG
101 TETYRPSSAS QCVAGIACAE IPVNQWPELI PQLVANVTNP NSTEHMKEST
151 LEAIGYICQD IDPEQLQDKS NEILTAIIQG MRKEEPSNNV K**LAATNALLN**
201 **SLEFTK**ANFD KESERHFIMQ VVCEATQCPD TRVRVAALQN LVKIMSLYYQ
251 YMETYMGPAL FAITIEAMKS DIDEVALQGI EFWSNVCDEE MDLAIEASEA
301 AEQGRPPEHT SKFYAK**GALQ YLVPILTQTL TKQDENDDDD DWNPCK**AAGV
351 CLMLLATCCE DDIVPHVLPF IKEHIKNPDW RYRDAAVMAF GCILEGPEPS
401 QLKPLVIQAM PTLIELMKDP SVVVRDTAAW TVGR**ICELLP EAAINDVYLA**
451 **PLLQCLIEGL SAEPR**VASNV CWAFSSLAEA AYEAADVADD QEEPATYCLS
501 SSFELIVQKL LETTDRPDGH QNNLRSSAYE SLMEIVKNSA KDCYPAVQKT
551 TLVIMERLQQ VLQMESHIQS TSDR**IQFNDL QSLLCATLQN VLR**KVQHQDA
601 LQISDVVMAS LLRMFQSTAG SGGVQEDALM AVSTLVEVLG GEFLKYMEAF
651 KPFLGIGLKN YAEYQVCLAA VGLVGDLCRA LQSNIIPFCD EVMQLLLENL
701 GNENVHRSVK PQILSVFGDI ALAIGGEFKK YLEVVLNTLQ QASQAQVDKS
751 DYDMVDYLNE LRESCLEAYT GIVQGLK**GDQ ENVHPDVMLV QPR**VEFILSF
801 IDHIAGDEDH TDGVVACAAG LIGDLCTAFG KDVLKLVEAR PMIHELLTEG
851 RRSKTNKAKT LATWATKELR KLKNQA

MS/MS Fragmentation of **WLAIDANAR**
Found in **IMB1_HUMAN** in **SwissProt**, Importin subunit beta-1 OS=Homo sapiens GN=KPNB1 PE=1 SV=2

Match to Query 390: 1028.539770 from(515.277161,2+) intensity(4362572.0000) rtinseconds(2784.4656) index(2013)
Title: 20171201_MNG_10uM_2.2831.2831.2 File:"20171201_MNG_10uM_2.raw", NativeID:"controllerType=0 controllerNumber=1 scan=2831"
Data file C:\Users\Mascott\Desktop\chiara cassisy\magnololo\MNGFISH\FISHING_04032017\20171201_MNG_10uM_2.mgf

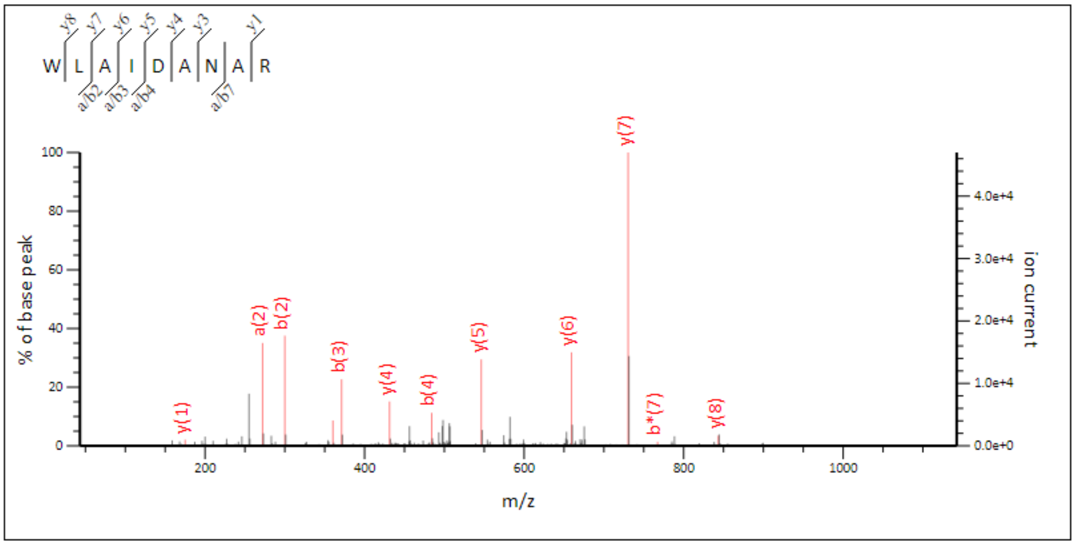

MS/MS Fragmentation of **IQFNDLQSLLCATLQNVLR**
Found in **IMB1_HUMAN** in **SwissProt**, Importin subunit beta-1 OS=Homo sapiens GN=KPNB1 PE=1 SV=2

Match to Query 2797: 2245.189062 from(1123.601807,2+) intensity(306473.5313) rtinseconds(3699.1077) index(3104)
Title: 20171201_MNG_10uM_2.3995.3995.2 File:"20171201_MNG_10uM_2.raw", NativeID:"controllerType=0 controllerNumber=1 scan=3995"
Data file C:\Users\Mascott\Desktop\chiara cassisy\magnololo\MNGFISH\FISHING_04032017\20171201_MNG_10uM_2.mgf

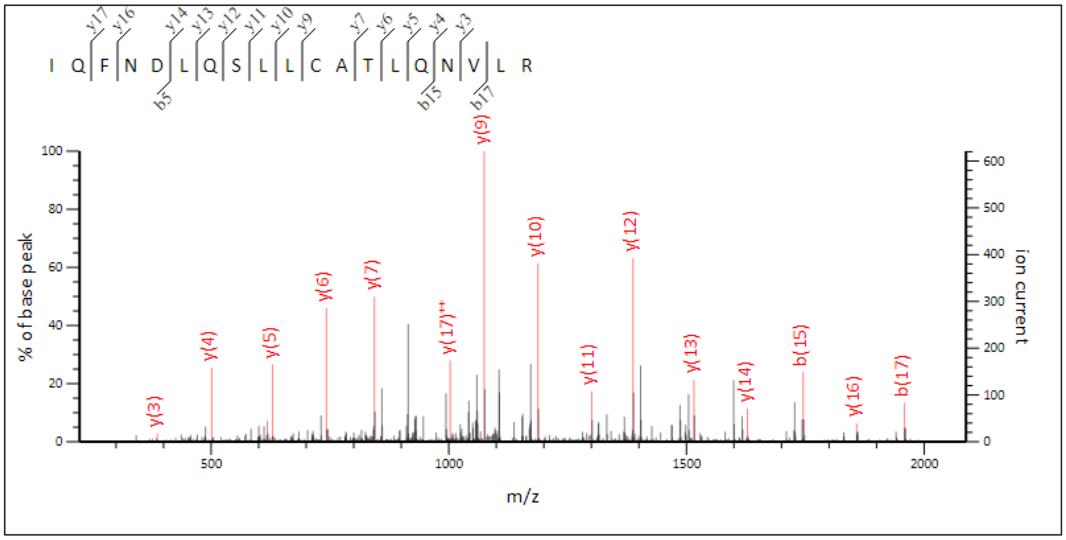

MS/MS Fragmentation of **QDENDDDDDWNPCK**
Found in **IMB1_HUMAN** in **SwissProt**, Importin subunit beta-1 OS=Homo sapiens GN=KPNB1 PE=1 SV=2

Match to Query 2267: 1764.623144 from(883.318848,2+) intensity(46095.1641) rtinseconds(2172.1965) index(1307)
Title: 20171201_MNG_10uM_2.2078.2078.2 File:"20171201_MNG_10uM_2.raw", NativeID:"controllerType=0 controllerNumber=1 scan=2078"
Data file C:\Users\Mascott\Desktop\chiara cassisy\magnololo\MNGFISH\FISHING_04032017\20171201_MNG_10uM_2.mgf

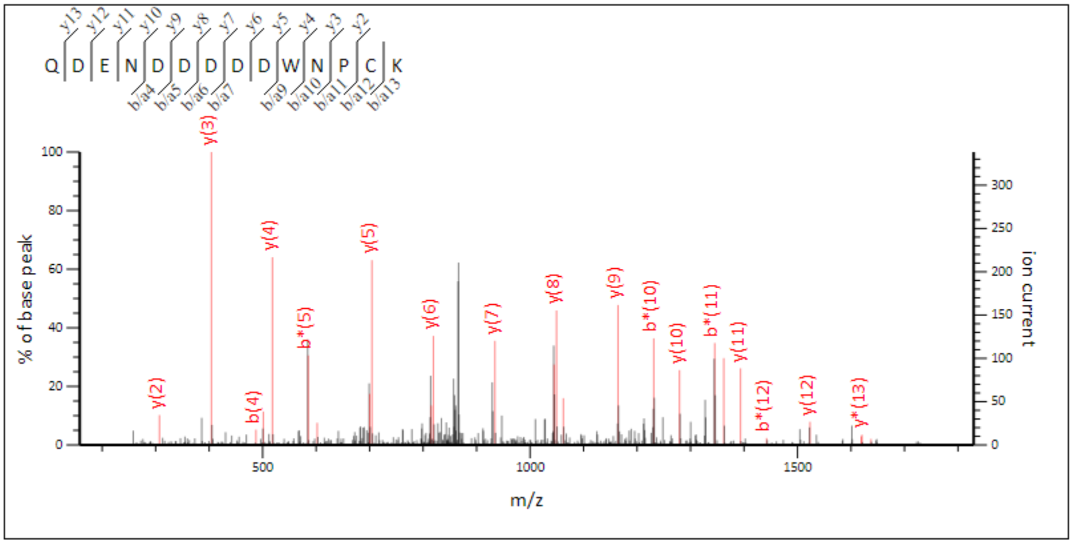

Exp 3:

MATRIX SCIENCE MASCOT Search Results

Protein View: IMB1_HUMAN

Importin subunit beta-1 OS=Homo sapiens GN=KPNB1 PE=1 SV=2

**Database:**	SwissProt
**Score:**	801
**Nominal mass (M_r):**	98420
**Calculated pI:**	4.68
**Taxonomy:**	Homo sapiens

Sequence similarity is available as an NCBI BLAST search of IMB1_HUMAN against nr.

Search parameters

**MS data file:**	C:\Users\Mascott\Desktop\chiara cassisy\magnololo\MNGFISH\FISHING_04032017\20171201_MNG_100uM_2.mgf
**Enzyme:**	Trypsin: cuts C-term side of KR unless next residue is P.
**Fixed modifications:**	Carbamidomethyl (C)
**Variable modifications:**	Oxidation (M), Phospho (ST)

Protein sequence coverage: 16%

Matched peptides shown in ***bold red***.

1	MELITILEKT	VSPDRLELEA	AQKFLER**AAV**	**ENLPTFLVEL**	**SR**VLANPGNS
51	QVARVAAGLQ	IKNSLTSKDP	DIKAQYQQR**W**	**LAIDANAR**RE	VKNYVLQTLG
101	TETYRPSSAS	QCVAGIACAE	IPVNQWPELI	PQLVANVTNP	NSTEHMKEST
151	LEAIGYICQD	IDPEQLQDKS	NEILTAIIQG	MRKEEPSNNV	K**LAATNALLN**
201	**SLEFTK**ANFD	KESERHFIMQ	VVCEATQCPD	TRVRVAALQN	LVKIMSLYYQ
251	YMETYMGPAL	FAITIEAMKS	DIDEVALQGI	EFWSNVCDEE	MDLAIEASEA
301	AEQGRPPEHT	SKFYAK**GALQ**	**YLVPILTQTL**	**TKQDENDDDD**	**DWNPCK**AAGV
351	CLMLLATCCE	DDIVPHVLPF	IKEHIKNPDW	RYRDAAVMAF	GCILEGPEPS
401	QLKPLVIQAM	PTLIELMKDP	SVVVRDTAAW	TVGRICELLP	EAAINDVYLA
451	PLLQCLIEGL	SAEPRVASNV	CWAFSSLAEA	AYEAADVADD	QEEPATYCLS
501	SSFELIVQKL	LETTDRPDGH	QNNLR**SSAYE**	**SLMEIVK**NSA	KDCYPAVQKT
551	TLVIMER**LQQ**	**VLQMESHIQS**	**TSDR**IQFNDL	QSLLCATLQN	VLRK**VQHQDA**
601	**LQISDVVMAS**	**LLR**MFQSTAG	SGGVQEDALM	AVSTLVEVLG	GEFLK**YMEAF**
651	**KPFLGIGLK**N	YAEYQVCLAA	VGLVGDLCRA	LQSNIIPFCD	EVMQLLLENL
701	GNENVHRSVK	PQILSVFGDI	ALAIGGEFKK	YLEVVLNTLQ	QASQAQVDKS
751	DYDMVDYLNE	LRESCLEAYT	GIVQGLK**GDQ**	**ENVHPDVMLV**	**QPR**VEFILSF
801	IDHIAGDEDH	TDGVVACAAG	LIGDLCTAFG	KDVLKLVEAR	PMIHELLTEG
851	RRSKTNKAKT	LATWATKELR	KLKNQA		

MS/MS Fragmentation of **AAVENLPTFLVELSR**
Found in **IMB1_HUMAN** in **SwissProt**, Importin subunit beta-1 OS=Homo sapiens GN=KPNB1 PE=1 SV=2

Match to Query 2189: 1657.905492 from(829.960022,2+) intensity(424368.2500) rtinseconds(3504.9404) index(2839)
Title: 20171201_MNG_100uM_2.3673.3673.2 File:"20171201_MNG_100uM_2.raw", NativeID:"controllerType=0 controllerNumber=1 scan=3673"
Data file C:\Users\Mascott\Desktop\chiara cassisy\magnololo\MNGFISH\FISHING_04032017\20171201_MNG_100uM_2.mgf

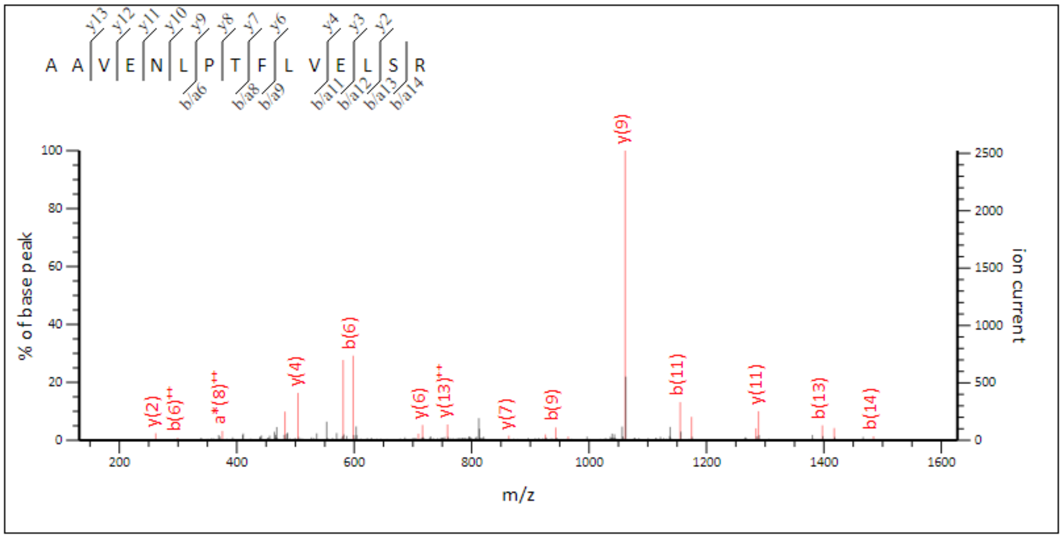

MS/MS Fragmentation of **QDENDDDDDWNPCK**
Found in **IMB1_HUMAN** in **SwissProt**, Importin subunit beta-1 OS=Homo sapiens GN=KPNB1 PE=1 SV=2

Match to Query 2298: 1764.621434 from(883.317993,2+) intensity(116297.6250) rtinseconds(2128.7131) index(1325)
Title: 20171201_MNG_100uM_2.2058.2058.2 File:"20171201_MNG_100uM_2.raw", NativeID:"controllerType=0 controllerNumber=1 scan=2
Data file C:\Users\Mascott\Desktop\chiara cassisy\magnololo\MNGFISH\FISHING_04032017\20171201_MNG_100uM_2.mgf

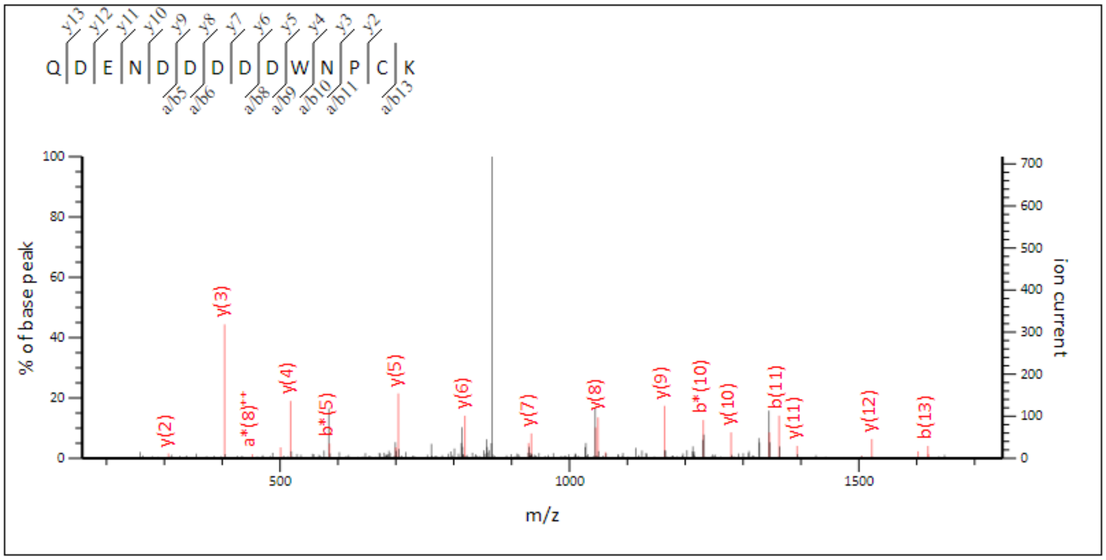

Exp 4:

MATRIX SCIENCE MASCOT Search Results

Protein View: IMB1_HUMAN

Importin subunit beta-1 OS=Homo sapiens GN=KPNB1 PE=1 SV=2

**Database:**	SwissProt
**Score:**	747
**Nominal mass (M_r):**	98420
**Calculated pI:**	4.68
**Taxonomy:**	Homo sapiens

Sequence similarity is available as an NCBI BLAST search of IMB1_HUMAN against nr.

Search parameters

**MS data file:**	C:\Users\Mascott\Desktop\chiara cassisy\magnololo\MNG
**Enzyme:**	Trypsin: cuts C-term side of KR unless next residue is P.
**Fixed modifications:**	Carbamidomethyl (C)
**Variable modifications:**	Oxidation (M), Phospho (ST)

Protein sequence coverage: 15%

Matched peptides shown in ***bold red***.

1	MELITILEK**T**	**VSPDRLELEA**	**AQK**FLER**AAV**	**ENLPTFLVEL**	**SR**VLANPGNS
51	QVARVAAGLQ	IKNSLTSKDP	DIKAQYQQR**W**	**LAIDANAR**RE	VKNYVLQTLG
101	TETYRPSSAS	QCVAGIACAE	IPVNQWPELI	PQLVANVTNP	NSTEHMKEST
151	LEAIGYICQD	IDPEQLQDK**S**	**NEILTAIIQG**	**MR**KEEPSNNV	K**LAATNALLN**
201	**SLEFTK**ANFD	KESERHFIMQ	VVCEATQCPD	TRVR**VAALQN**	**LVK**IMSLYYQ
251	YMETYMGPAL	FAITIEAMKS	DIDEVALQGI	EFWSNVCDEE	MDLAIEASEA
301	AEQGRPPEHT	SKFYAKGALQ	YLVPILTQTL	TKQDENDDDD	DWNPCKAAGV
351	CLMLLATCCE	DDIVPHVLPF	IKEHIKNPDW	RYRDAAVMAF	GCILEGPEPS
401	QLKPLVIQAM	PTLIELMKDP	SVVVR**DTAAW**	**TVGR**ICELLP	EAAINDVYLA
451	PLLQCLIEGL	SAEPRVASNV	CWAFSSLAEA	AYEAADVADD	QEEPATYCLS
501	SSFELIVQK**L**	**LETTDRPDGH**	**QNNLR**SSAYE	SLMEIVKNSA	KDCYPAVQK**T**
551	**TLVIMERLQQ**	**VLQMESHIQS**	**TSDR**IQFNDL	QSLLCATLQN	VLRKVQHQDA
601	LQISDVVMAS	LLRMFQSTAG	SGGVQEDALM	AVSTLVEVLG	GEFLKYMEAF
651	KPFLGIGLKN	YAEYQVCLAA	VGLVGDLCRA	LQSNIIPFCD	EVMQLLLENL
701	GNENVHRSVK	PQILSVFGDI	ALAIGGEFKK	YLEVVLNTLQ	QASQAQVDK**S**
751	**DYDMVDYLNE**	**LR**ESCLEAYT	GIVQGLKGDQ	ENVHPDVMLV	QPRVEFILSF
801	IDHIAGDEDH	TDGVVACAAG	LIGDLCTAFG	KDVLKLVEAR	PMIHELLTEG
851	RRSKTNKAKT	LATWATKELR	KLKNQA		

MS/MS Fragmentation of **TVSPDRLELEAAQK**
Found in **IMB1_HUMAN** in **SwissProt**, Importin subunit beta-1 OS=Homo sapiens GN=KPNB1 PE=1 SV=2

Match to Query 2680: 1555.819676 from(778.917114,2+) intensity(418387.9375) rtinseconds(2379.6228) index(1799)
Title: 20171214_MNGRESINAMNG_2.2379.2379.2 File:"20171214_MNGRESINAMNG_2.raw", NativeID:"controllerType=0 controllerNumber=1 scan=237
Data file C:\Users\Mascott\Desktop\chiara cassisy\magnololo\MNGFISH\COMPETITIVE_ELUTION_20171712\20171214_MNGRESINAMNG_2.mgf

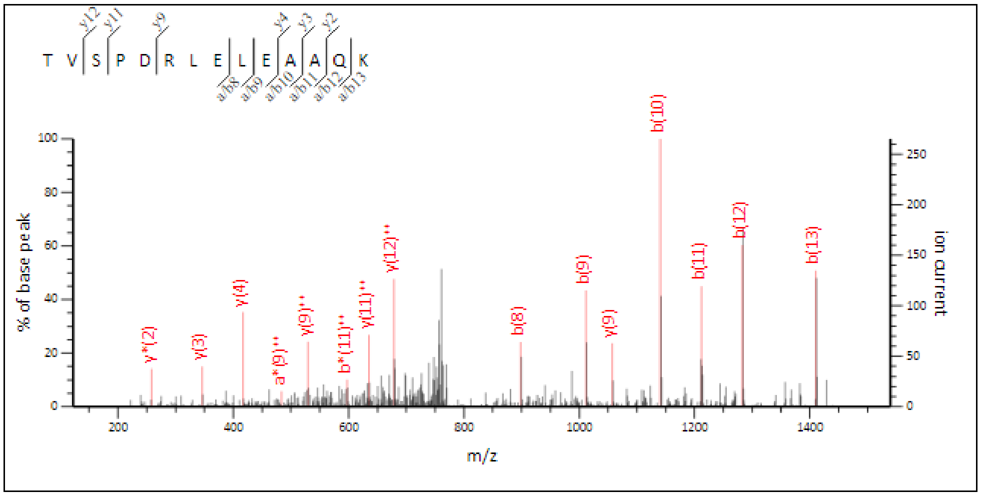

MS/MS Fragmentation of **AAVENLPTFLVELSR**
Found in **IMB1_HUMAN** in **SwissProt**, Importin subunit beta-1 OS=Homo sapiens GN=KPNB1 PE=1 SV=2

Match to Query 2793: 1657.903906 from(829.959229,2+) intensity(102956.1250) rtinseconds(3505.2868) index(2977)
Title: 20171214_MNGRESINAMNG_2.3636.3636.2 File:"20171214_MNGRESINAMNG_2.raw", NativeID:"controllerType=0 controllerNumber=1 scan=3636"
Data file C:\Users\Mascott\Desktop\chiara cassisy\magnololo\MNGFISH\COMPETITIVE_ELUTION_20170712\20171214_MNGRESINAMNG_2.mgf

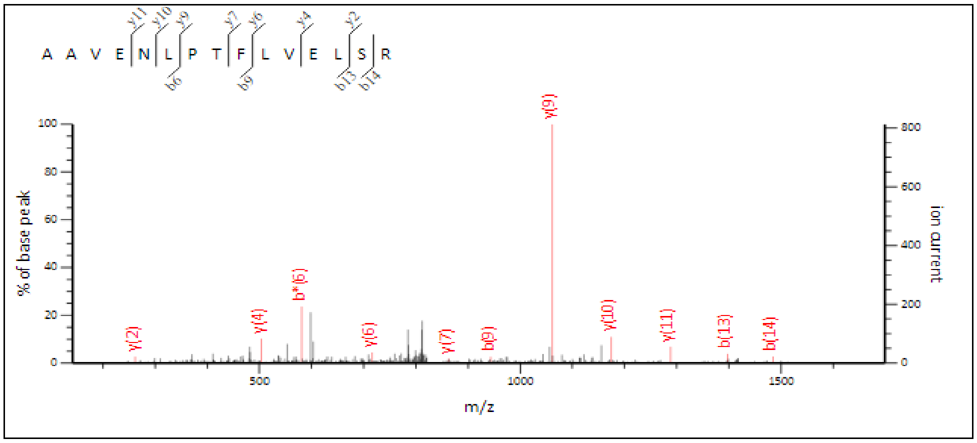

MS/MS Fragmentation of **WLAIDANAR**
Found in **IMB1_HUMAN** in **SwissProt**, Importin subunit beta-1 OS=Homo sapiens GN=KPNB1 PE=1 SV=2

Match to Query 798: 1028.539404 from(515.276978,2+) intensity(1933732.7500) rtinseconds(2855.2781) index(2303)
Title: 20171214_MNGRESINAMNG_2.2917.2917.2 File:"20171214_MNGRESINAMNG_2.raw", NativeID:"controllerType=0 controllerNumber=1 scan=2917"
Data file C:\Users\Mascott\Desktop\chiara cassisy\magnololo\MNGFISH\COMPETITIVE_ELUTION_20170712\20171214_MNGRESINAMNG_2.mgf

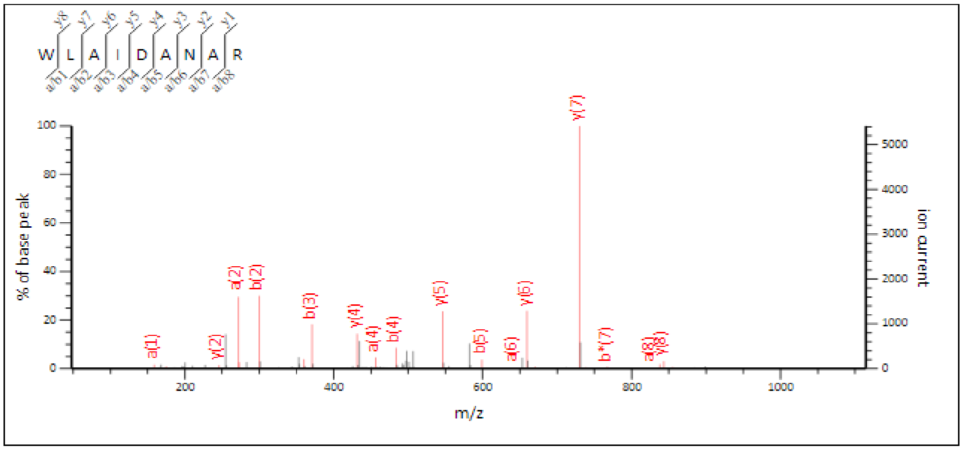

MS/MS Fragmentation of **VAALQNLVK**
Found in **IMB1_HUMAN** in **SwissProt**, Importin subunit beta-1 OS=Homo sapiens GN=KPNB1 PE=1 SV=2

Match to Query 267: 954.585668 from(478.300110,2+) intensity(4573291.5000) rtinseconds(2646.8936) index(2087)
Title: 20171214_MNGRESINAMNG_2.2687.2687.2 File:"20171214_MNGRESINAMNG_2.raw", NativeID:"controllerType=0 controllerNumber=1 scan=2687"
Data file C:\Users\Mascott\Desktop\chiara cassisy\magnololo\MNGFISH\COMPETITIVE_ELUTION_20170712\20171214_MNGRESINAMNG_2.mgf

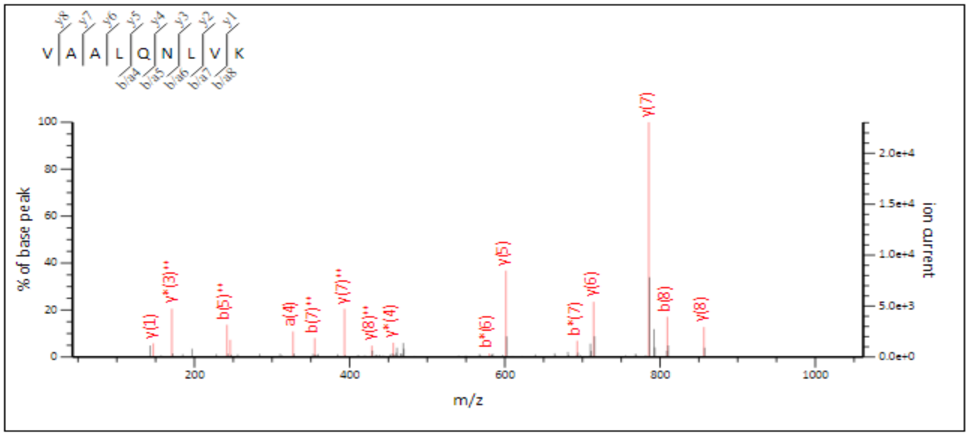

Supplementary Figure 3. Example of MASCOT Search Results for IMP-β1 identification followed by tandem mass spectra of different peptides of the protein, in four independent experiments.

5. Molecular docking analysis of the Honokiol/Importin β-1 complex

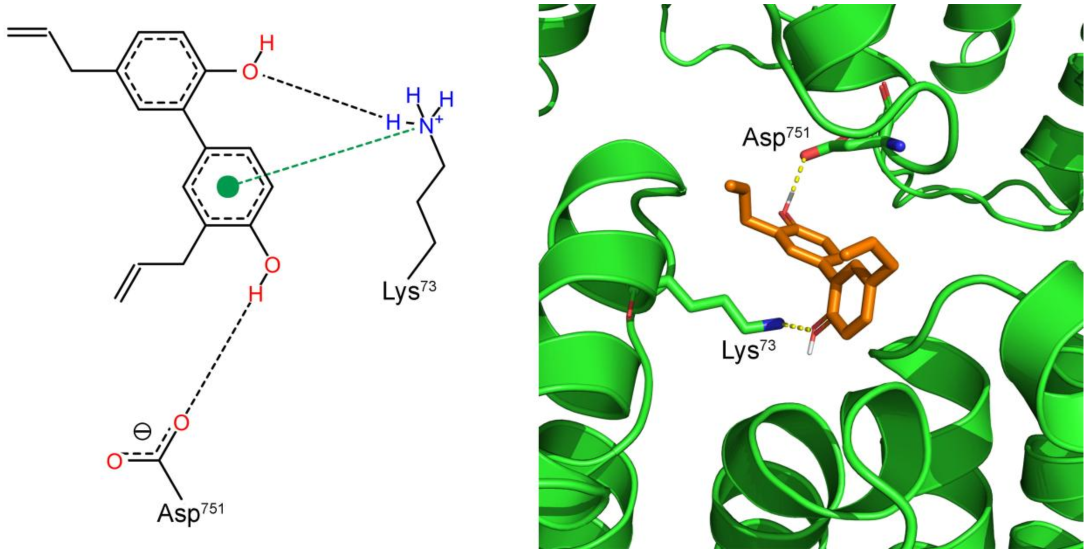

Supplementary Figure 4: Best feasible binding pose predicted by the molecular docking analysis between Importin β-1 and honokiol. Residues involved in the complex formation are shown in details. The predicted equilibrium dissociation constant ($K_{D,pred}$) related to this binding site was of 0.22 ± 0.05 µM.

6. MTT assay for measuring cell viability in presence or in absence of MNG

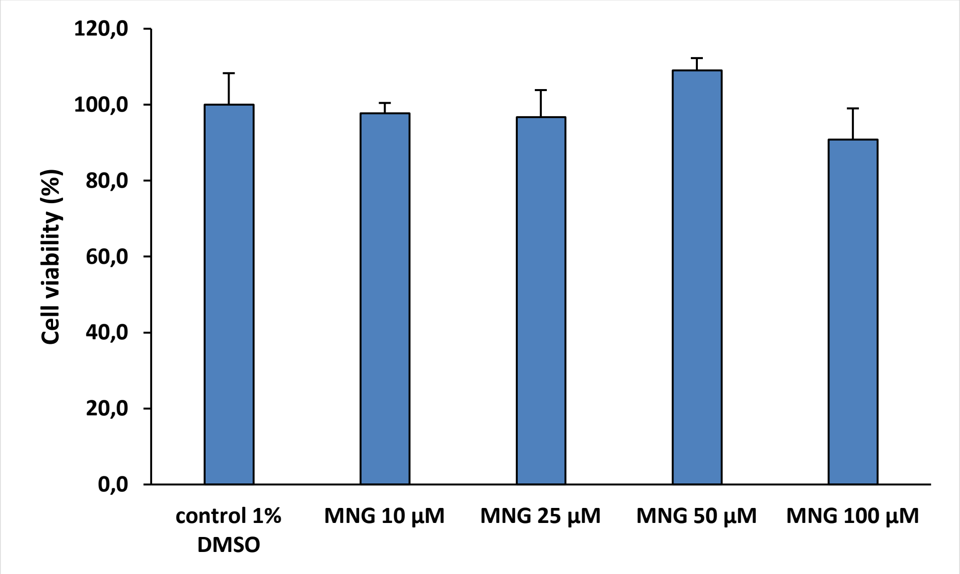

Supplementary Figure 5. The viability of cells was determined using the MTT [3-(4,5-dimethylthiazol-2-yl)-2,5-diphenyltetrazolium bromide] assay. $5x10^4$ HeLa cells/well were seeded in 96 well plate and, after 24 hours, treated in quadruplicate with increasing concentration of MNG (0, 10, 25, 50 and 100 µM) in 1% DMSO as vehicle. Cells treated only with 1% DMSO were used as control. After 16 hours of treatment, 10 µl of MTT solution was added in each well and the cells were incubated for 1 h at 37 °C and 5% CO_2 in the dark. Then, the formazan crystals were dissolved in DMSO (100 µL/well) and the absorbance was recorded at a wavelength of 550 nm with reference at 620 nm.
